# Characterizing Acupuncture Stimuli Using Brain Imaging with fMRI - A Systematic Review and Meta-Analysis of the Literature

**DOI:** 10.1371/journal.pone.0032960

**Published:** 2012-04-09

**Authors:** Wenjing Huang, Daniel Pach, Vitaly Napadow, Kyungmo Park, Xiangyu Long, Jane Neumann, Yumi Maeda, Till Nierhaus, Fanrong Liang, Claudia M. Witt

**Affiliations:** 1 Institute for Social Medicine, Epidemiology and Health Economics, Charité University Medical Center, Berlin, Germany; 2 Chengdu University of Traditional Chinese Medicine, Chengdu, China; 3 Center for Integrative Medicine, University of Maryland School of Medicine, Baltimore, Maryland, United States of America; 4 Athinoula A. Martinos Center for Biomedical Imaging, Department of Radiology, Massachusetts General Hospital, Charlestown, Massachusetts, United States of America; 5 Department of Radiology, Logan College of Chiropractic, Chesterfield, Missouri, United States of America; 6 Department of Biomedical Engineering, Kyung Hee University, Yongin, Republic of Korea; 7 Berlin NeuroImaging Center and Department Neurology, Charité, Berlin, Germany; 8 Max Planck Institute for Human Cognitive and Brain Sciences, Leipzig, Germany; 9 Leipzig University Medical Center, IFB Adiposity Diseases, Leipzig, Germany; The University of Melbourne, Australia

## Abstract

**Background:**

The mechanisms of action underlying acupuncture, including acupuncture point specificity, are not well understood. In the previous decade, an increasing number of studies have applied fMRI to investigate brain response to acupuncture stimulation. Our aim was to provide a systematic overview of acupuncture fMRI research considering the following aspects: 1) differences between verum and sham acupuncture, 2) differences due to various methods of acupuncture manipulation, 3) differences between patients and healthy volunteers, 4) differences between different acupuncture points.

**Methodology/Principal Findings:**

We systematically searched English, Chinese, Korean and Japanese databases for literature published from the earliest available up until September 2009, without any language restrictions. We included all studies using fMRI to investigate the effect of acupuncture on the human brain (at least one group that received needle-based acupuncture). 779 papers were identified, 149 met the inclusion criteria for the descriptive analysis, and 34 were eligible for the meta-analyses. From a descriptive perspective, multiple studies reported that acupuncture modulates activity within specific brain areas, including somatosensory cortices, limbic system, basal ganglia, brain stem, and cerebellum. Meta-analyses for verum acupuncture stimuli confirmed brain activity within many of the regions mentioned above. Differences between verum and sham acupuncture were noted in brain response in middle cingulate, while some heterogeneity was noted for other regions depending on how such meta-analyses were performed, such as sensorimotor cortices, limbic regions, and cerebellum.

**Conclusions:**

Brain response to acupuncture stimuli encompasses a broad network of regions consistent with not just somatosensory, but also affective and cognitive processing. While the results were heterogeneous, from a descriptive perspective most studies suggest that acupuncture can modulate the activity within specific brain areas, and the evidence based on meta-analyses confirmed some of these results. More high quality studies with more transparent methodology are needed to improve the consistency amongst different studies.

## Introduction

Acupuncture is a therapy of inserting and manipulating fine filiform needles into specific body locations (acupuncture points) to treat diseases. Acupuncture is an ancient Chinese treatment that has been systematically used for over 2000 years [1]. Currently, acupuncture is used widely all over the world, but its biological mechanism is not well understood. From a neurophysiological aspect acupuncture can be regarded as a complex somatosensory stimulation [2]. Although the clinical effect of acupuncture is generally accepted for certain diagnoses [3], such as knee pain, low back pain etc., there exists controversy regarding the specific effect of acupuncture, especially for the specificity of acupuncture points and meridians. In clinical studies large effects produced by sham acupuncture were observed [4]–[6].

Interest in investigating acupuncture mechanisms with imaging techniques has been growing since the mid 1990 s [7], [8]. Positron emission tomography (PET), single photon emission computed tomography (SPECT), and magnetic resonance imaging (MRI) have been used and, there is also interest in electro-encephalography (EEG). Functional MRI (fMRI), investigating the hemodynamic blood oxygenation level dependent (BOLD) effect, has come to dominate the brain mapping field due to its minimal invasiveness, lack of radiation exposure, excellent spatial resolution and relatively wide availability.

In the previous decade, an increasing number of studies applied fMRI to investigate acupuncture stimulation. The aim of this review was to give a systematic overview about the fMRI research on acupuncture regarding the following four aspects: 1) differences between verum and sham acupuncture, 2) differences due to various methods of acupuncture manipulation, 3) differences between patients and healthy volunteers, 4) differences between different acupuncture points.

## Methods

The search strategy, research questions, inclusion and exclusion criteria and data extraction and analysis were predefined in our protocol. During the study, the database search was extended for the Japanese and Korean databases.

### Searching

We searched the following sources:

1.PubMed (1948–2009.09) 2.EMBASE (1980–2009.09) 3.CNKI (China National Knowledge Infrastructure) (1915–2009.09) 4.Japanese Ichushi-Web (1983–2009.09) 5.Korean NDSL (National Digital Science Links) (1946–2009.09); KTKP (Korean Traditional Knowledge Portal) (1997–2008)

We searched these databases in the appropriate language using the following MeSH terms and search strategies:

English: 1.fMRI; 2.Functional MRI; 3.MRI, Functional; 4.Magnetic Resonance Imaging, Functional; 5.acupuncture; 6.#1 or #2 or #3 or #4; 7.#5 and #6;

Chinese: 1.针刺(acupuncture); 2.磁共振成像(Magnetic Resonance Imaging); 3.#1 and #2

Japanese: 1.1.鍼(acupuncture); 2.機能的磁気共鳴画像法 (Functional Magnetic Resonance Imaging); 3.#1 and #2

Korean: 1.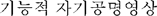 (fMRI, functional Magnetic Resonance Imaging); 2.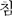 (acupuncture); 3.#1 and #2

We screened the bibliographies of identified trials and reviewed articles for further potentially relevant publications.

### Selection

In this review we included all studies using fMRI to investigate the effect of acupuncture on the human brain. Each study had to have at least one group, which received an intervention with any type of needle-based acupuncture. We included trials on healthy volunteers as well as patients and all types of needle acupuncture were accepted. There were no language restrictions and no limitations on outcome measures. Reviews, editorials and trials on animals were excluded.

The available abstracts of all identified references were screened and we excluded all citations that clearly did not fit the inclusion criteria. Full copies of all remaining articles and those references without available abstracts were obtained. Subsequently the three researchers (WJH: Pubmed, Embase and CNKI, KP: Korean databases, YM: Japanese databases) screened the full texts and assessed whether these trials met the inclusion criteria.

In the meta-analysis, we included studies investigating only verum acupuncture or both verum and sham acupuncture by fMRI using whole brain acquisition. Studies were excluded if 1) the number of study participants was less than five; 2) results were not reported as 3-dimensional coordinates in standard stereotactic space; 3) only the results from regions of interest (ROI) were reported or 4) only single subject data instead of group data were reported.

### Data extraction and analysis

The three researchers (WJH: Pubmed, Embase and CNKI, KP: Korean databases, YM: Japanese databases) extracted the data for all descriptive information from the publications, namely published journals, language, study place, study type, subjects, handedness, objective, interventions, control groups, block-design, fMRI device type, software for fMRI data analysis, sample size, and results. The extracted data were discussed with three supervisors (CW, DP and VN). Any inconsistencies were discussed and reconsidered until consensus was reached.

Results were structured according to the four research questions. Studies that matched multiple research questions were displayed more than once, but only with the part of the study relevant to the respective research question.

Furthermore, one figure for different acupuncture points from publications in Talairach coordinates was generated by one author (XYL) using Analysis of Functional NeuroImages (AFNI, http://afni.nimh.nih.gov) and MRIcron software (http://www.cabiatl.com/mricro). The anatomical image was generated using MRIcron software.

The meta-analyses were conducted (JN, XYL, WJH) in Talairach space, using the activation likelihood estimation technique (ALE) implemented in GingerALE 2.1.1 software [9]–[11]. This technique assesses the convergence between activation foci from different experiments. Prior to the analysis, coordinates reported in MNI (Montreal Neurological Institute) space were converted to Talairach anatomical space using the Lancaster transform [12]. For each experiment, every reported activation maximum was modeled by a 3-dimensional Gaussian probability distribution centered at the given coordinate. The width of the Gaussian probability distribution was determined individually for each experiment based on empirical estimates of between-subject variability, taking into account the number of subjects in each experiment [9]. Voxel-wise ALE scores were calculated from the union of the Gaussian probability distributions within and across experiments. In a random effects analysis, ALE scores were tested against a null hypothesis of random distribution across the brain, thereby identifying those regions where empirical ALE values were higher than could be expected by chance. Resulting ALE maps were thresholded at p<0.05 (corrected for multiple comparisons by False Discovery Rate). The minimum cluster volume was chosen to exceed the number of voxels corresponding to 5% possible false positives. The contrast studies analysis (subtraction analysis which compares two ALE maps) was performed with randomization testing with 10,000 permutations. As there exists no correction for multiple comparison with this approach, the threshold was set at p<0.05 (uncorrected) with a min. cluster size = 200 mm^3^ [13].

ALE maps were computed for the following statistical comparisons. From all studies included in the meta-analysis: 1a) greater activation of verum acupuncture points compared to baseline (verum>rest), 1b) greater deactivation of verum acupuncture points compared to baseline (rest>verum). From the studies which provided direct contrasts between verum and sham acupuncture: 2a) greater activation from verum than sham acupuncture (or greater deactivation for sham, i.e. verum>sham), 2b) greater deactivation from verum than sham acupuncture (or greater activation for sham, i.e. sham>verum). From the studies which had both verum and sham acupuncture groups: 3a) greater activation of verum acupuncture points than baseline (verum>rest), 3b) greater deactivation of verum acupuncture points than baseline (rest>verum), 3c) greater activation of sham acupuncture points than baseline (sham>rest), 3d) greater deactivation of sham acupuncture points than baseline (rest>sham), 3e) comparison ALE map of greater activation of verum than sham acupuncture relative to rest (“verum>rest” - “sham>rest”), 3f) comparison ALE map of greater deactivation of verum than sham acupuncture relative to rest (“rest>verum” - “rest>sham”).

## Results

### Study characteristics

**Figure 1:**
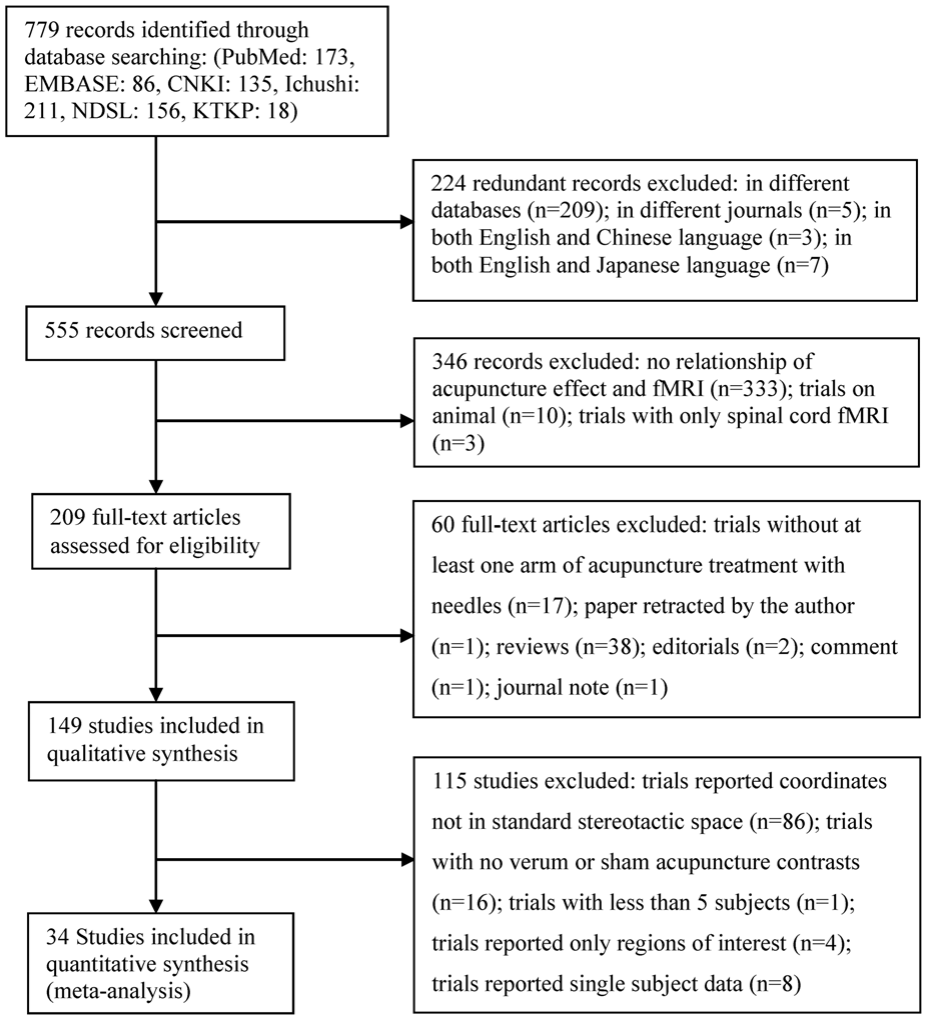
Flow of information through the different phases of the systematic review.

**Figure 2:**
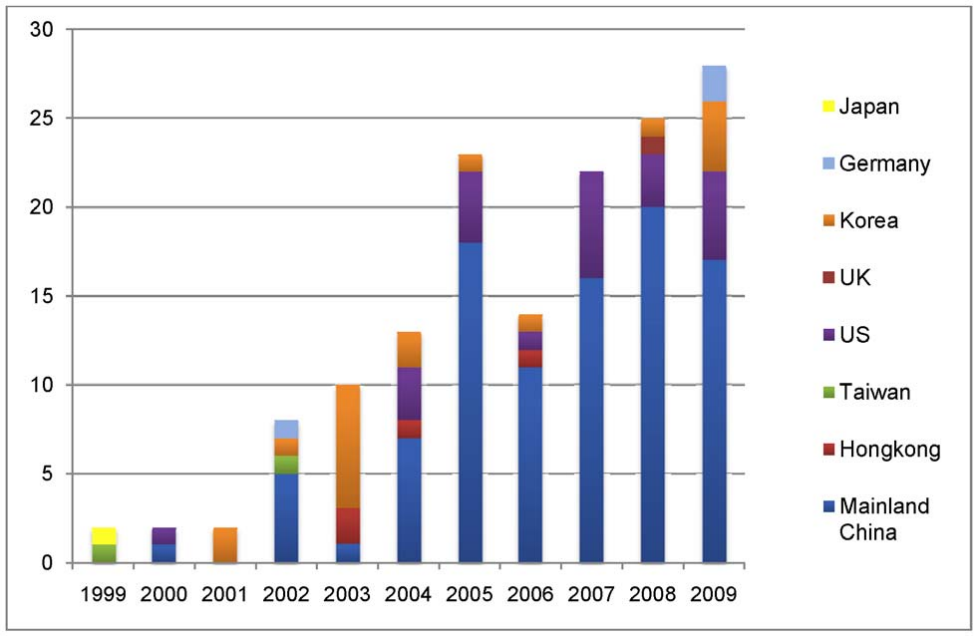
Number of publications on acupuncture and fMRI identified in the last 11 years.

The 149 studies were published between 1999 and 2009 (trial flow see Figure 1), Figure 2 shows the number of publications per year in corresponding countries in the last 11 years. Most of the studies were performed in China, US and Korea and predominantly published in Chinese and English (50.3% Chinese, 38.9% English, 9.4% Korean, 0.7% German and 0.7% Japanese). The median number of subjects per study was 17 (min. 1 to max. 67), and the total number of all studies included 2469 subjects. 24 studies reported parallel group randomized trials. 128 studies were on healthy volunteers, 13 studies on patients, 8 studies on the comparison of patients and healthy volunteers. Most of the trials applied a block design for fMRI data acquisition, with a time range for each block of 8 sec to 6 min, and the number of blocks ranged from one to 12 blocks. 105 studies included right-handed subjects while only 3 studies included also left-handed subjects. 34 studies were included in the meta-analyses.

### Descriptive findings of differences between verum and sham acupuncture

51 publications explored four kinds of sham acupuncture including a) a placebo needle (Streitberger needle [14]: with a blunt tip, which when it touches the skin causes a pricking sensation for the patient, simulating the puncturing of the skin. The needle moves inside the handle, and appears to be shortened.); b) needling at non-acupuncture points in close proximity to acupuncture points; c) needling at non-acupuncture points distant to acupuncture points; d) cutaneous stimulation at the same acupuncture points or sham point/area (Table S1). Two of the studies [15], [16] are referenced more than once in the table because of the different sham acupuncture methods evaluated in these studies. The studies included mainly healthy volunteers, but four publications [17]–[20] included patients with Parkinson's disease or stroke.

#### A placebo needle: Streitberger Needle

The four studies which compared verum acupuncture with the Streitberger Needle were all from the US and showed heterogeneous results [16], [19], [21], [22]. Yoo et al. [16] found more activation associated with verum acupuncture in the somatosensory areas and motor areas. Dougherty et al. [21] reported that acupuncture produced more activation in the medial orbitofrontal cortex and more deactivation in brainstem and insula, while the Streitberger needle showed higher activation in the language area (Wernicke), pons, operculum and insula. According to Deng et al. [22] verum acupuncture resulted in more activation in insula and operculum compared to the Streitberger needle placed at a non-acupuncture point. A study with stroke patients [19] (scan during passive finger movement pre and -post 10 weeks treatment of verum acupuncture or the Streitberger placebo needle) showed a trend toward a greater maximum activation change in the motor cortical area for the verum acupuncture group.

#### Acupuncture at non-acupuncture points in close proximity to acupuncture points

Two third (64%) [15], [23]–[37] of 25 studies showed that acupuncture treatments were associated with more activation, mainly in the somatosensory areas, motor areas, basal ganglia, cerebellum, limbic system and higher cognitive areas (e.g. prefrontal cortex). Three studies [28], [37], [38] showed also more deactivations in the limbic system in response to acupuncture. In contrast, one study [39] found greater activation in the supplementary motor area in response to sham acupuncture. Five other studies [40]–[44] found no significant difference between verum and sham acupuncture. One experiment was analyzed twice [45], [46] and came to different results.

#### Acupuncture at non-acupuncture points distant to acupuncture points

Of six studies, two studies [47], [48] showed no differences between verum and sham acupuncture. Four studies [49]–[52] showed more activation associated with acupuncture in the somatosensory areas, brainstem, basal ganglia, higher cognitive areas and part of the limbic system (hypothalamus, nucleus accumbens), and one study [52] showed more activation associated with sham acupuncture in the motor area and operculum. Verum acupuncture showed also more deactivation in part of the limbic system (amygdala, hippocampus, cingulate gyrus/cortex) [47], [52]. In addition, Napadow et al. [51] found that both verum and sham acupuncture showed linearly decreasing activation over repeated stimulus blocks in the sensorimotor areas, while verum acupuncture produced bimodal activity in a limbic midbrain region - activation in early blocks, but deactivation in later stimulus blocks.

#### Cutaneous stimulation at the same acupuncture point or sham point/area

There are 18 studies (15 on healthy volunteers). Only one study [16] on healthy volunteers found greater activation in the somatosensory area during verum acupuncture, whereas in four studies [53]–[56] somatosensory activation was greater with cutaneous stimulation. For motor areas and higher cognitive areas, five studies [15], [16], [55], [57], [58] showed that acupuncture was associated with more activation. For brainstem, basal ganglia, cerebellum and limbic system the results were complex or contradictory: in the basal ganglia, brainstem and cerebellum, two studies [53], [59] found that acupuncture was associated with more deactivation while three other studies [15], [57], [60] found acupuncture associated with more activation; thalamus and insula [15], [16], [54], [58] were activated more while hypothalamus, hippocampus, amygdala and temporal pole [53], [54], [58], [59] were deactivated more by acupuncture. In addition, when eliciting deqi, Hui et al. [53] found extensive deactivation in the cerebrum, brainstem and cerebellum, while eliciting deqi mixed with pain, activation was the predominant pattern. Five Chinese studies [61]–[65] found almost no significant differences between verum and sham, though two of them found greater activation intensity in the cerebellum or parietal lobe for verum acupuncture [61], [62]. Among the three publications on patients, Schockert et al. [20] found more activation in the motor area on stroke patients during acupuncture while Li et al. [17] found more activation in the somatosensory and motor areas with a control, brushing stimulation on stroke patients. In patients with Parkinson's disease Chae et al. [18] showed that acupuncture was associated with more activation than covert cutaneous stimulation in the motor area, basal ganglia, visual and higher cognitive area; and more activation in the motor, visual, higher cognitive areas and limbic system, compared to overt cutaneous stimulation.

### Descriptive findings of differences due to various methods of acupuncture manipulation

Manipulation methods can differ in the depth of needling, forms of needle stimulation (e.g. manual versus electrical), intensity of stimulation, and stimulus timing parameters (e.g. duration, frequency, etc.). Here, we summarized the results from those studies comparing different methods of manipulation at acupuncture points in healthy volunteers (see Table 1). Two of the studies [58], [66] are displayed more than once in the table as they explored multiple comparisons.

**Table 1:** Descriptive analysis of differences due to various methods of acupuncture manipulation.

Author (year)	Language	Studyplace	Study design	Case NO.	Group NO.	Intervention	Control	Points	Statistic	Group differences which result in more activation	Group differences which result in more deactivation
**a) Comparison of Different Needling Depths**											
Li et al. 2000	C	CN	NCT	26	2	MA (muscle layer)	MA (round tip, non-penetrating, stimulating between the epidermis and dermis)	ST36, ST32	NA	NSD	NSD
MacPherson et al. 2008	E	UK	RIO, PB	17	2	MA (8–12 mm)	MA (1–2 mm)	LI4 (R)	Y	NSD	NSD
Zhang et al. 2007	E	CN	NCT,PB	12	2	EA (2–3 cm)	EA (subcutaneous)	GB34, GB39 (L)	Y	*EA (2–3 cm)>EA* (*subcutaneous):* C*on. SI, SII, MC, ant. CingC, IN, Th, H, OC, Ce; Bil. PFG, Cau and P*	NSD
Wu et al. 1999	E	CN	Semi-RIO, PB	18	2	MA (2 cm)	MA (1 mm)	ST36 (L)	NA	1) MA (2 cm)>MA (1 mm): Con.Hyp, Nac; 2) MA (1 mm)>MA (2 cm): SI, Th, ant. CingC (BA 32, 34); Con. SMA; Bil. Fop (BA44 and SMA), PO (BA40)	1)MA (2 cm)>MA (1 mm): Bil. Ant. CingC (rostral part, BA 24B), Ipsi. OG, BG, Con. Amyg, H
**b) Comparison of Electro-acupuncture vs. Manual Acupuncture**											
Kong et al. 2002	E	CN	RIO	11	2	MA (3 Hz, 180 rpm)	EA (3 Hz)	LI4 (L)	Y	*EA>MA: Con. preCG; SII (CO, PO); Ipsi. Put/In*	*1) MA>EA: Con. STG and Put/IN, post. Cing, STG and Ipsi. LN/In; 2) EA>MA: Con. preCun*
Li et al. 2003	E	CN	NCT	20	3	MA	1) EA (2 Hz); 2) EA (20 Hz)	BL60, 65, 66, 67 (R)	Y	NSD	*MA>2 EA groups: Bil. Cun(BA18), TTG (BA41), MFG (BA46)*
Napadow et al. 2005	E	US	NCT, PB	13	3	MA (ERRM, 1 Hz)	1) EA (2 Hz); 2) EA (100 Hz)	ST36 (L)	Y	*1) EA 2 Hz >MA: SI, Con. Cing-am, NRP; 2) EA 100 Hz>MA: SI, Con. Cing-am*	*EA>MA: septal area*
**c) Comparison of Different Frequencies of Electro-acupuncture Stimulation**											
Li et al. 2003	E	CN	NCT	20	2	EA (2 Hz)	EA (20 Hz)	BL60, 65, 66, 67 (R)	Y	NSD	NSD
Napadow et al. 2005	E	US	NCT, PB	13	2	EA (2 Hz)	EA (100 Hz)	ST36 (L)	Y	*EA 2 Hz>EA 100 Hz: NRP*	NA
**d) Comparison of Different Intensities of Manual Acupuncture Stimulation**											
Li et al 2006	E	CN	RCT/P	18	3	MA (30 s)	1) MA (60 s); 2) MA (180 s)	LI4 (R)	Y	*1) 60 s>30 s: Ipsi. IFG,ITG; 2) 180 s>60 s: Bil. Tpole, Ce, OL; 3) 180 s>30 s: Bil. Tpole, Ce, IPL*	*1) 60 s>30 s: Bil. OG, Ipsi. TL, P; 2) 180 s>60 s: Bil. dlPFC, MEFG; 3) 180 s>30 s: Bil. dlPFC, MEFG*
Gareus et al. 2002	E	DE	NCT	21	2	MA (twisting)+visual stimu (Bil.)	MA (no stimu)+visual stimu (L)	GB37	NA	1) MA(twisting+visual)>MA(no stimu+visual): IN, PO, PTC, IPL, supCol, Cun, MOG, CingG	NA
Hu et al. 2005	C	CN	RCT	19	3	MA(twirling, 120–200 rpm) (Bil.)	1) MA (no stimu)+visual stimu (L); 2) MA (no stimu)+visual stimu (Bil.)	GB37, LR3	Y	MA>MA (no stimuli)+visual stimuli (L/Bil.): V1	NA
Fang et al. 2004	E	CN	RIO, PB	15	2	MA (ERRM, rotating, 2 Hz)	MA (no stimu)	LR3, GB40 (L)	Y	*MA(rotaing)>MA(no stimu): Bil. SII; Ipsi. FOP(BA10), Ce; Con. Th*	NA
Cheng et al. 2009	C	CN	NCT	12	2	MA (rotating, 1.5 Hz)	MA (no stimu)	KI3 (R)	Y	*MA(rotaing)>MA(no stimu): Ipsi. STG(BA22); Con. MFG(BA46),IFG(BA45), IPL(BA40); Bil. postCG(BA2,3)*	NA
Gong et al. 2003	C	CN	NCT, PB, OB	64	2	MA (with deqi, 1 cun deep, thrusting and lifting at 0.3–0.5 cun)	MA (no deqi, 0.4 cun deep, thrusting and lifting at 0.1–0.2 cun)	ST36, ST39 (R)	Y	*deqi>no deqi: Bil. CingC, IN, upper wall of latS, Con. postCG*	NA

Words in italics means statistically significant;

Amyg = Amygdala, ant. = anterior, BA = Brodmann area, BG = basal gyrus, Bil. = bilateral, C = Chinese, Cau = caudate nucleus, Ce = cerebellum, Cing = cingulate, Cing-am = anterior middle cingulate, CingC = cingulate cortex, CingG = cingulate gyrus, CN = China, CO = central operculum, Con. = contralateral, Cun = cuneus, DE = Germany, dlPFC = dorsolateral prefrontal cortex, E = English, EA =  electro-acupuncture, ERRM = even reinforcing and reducing method, Fop = frontal operculum, H = hippocampus, Hyp = hypothalamus, IFG = inferior frontal gyrus, IN = insula, Ipsi = ipsilateral, IPL = inferior parietal lobule, ITG = inferior temporal gyrus, L = left, latS = lateral sulcus, LN = lenticular nucleus, MA =  manual acupuncture, MC = motor cortex, MEFG = medial frontal gyrus, MFG = middle frontal gyrus, MOG = middle occipital gyrus, NA =  information unavailable, Nac = nucleus accumbens, NCT =  non-randomized controlled trial, NRP = nucleus raphe pontis, NSD =  non statistically different, OB = observer blinded, OC = occipital cortex, OG = orbital gyrus, OL = occipital lobe, P = pons, PB =  patient blinded, PFG = prefrontal gyrus, PO = parietal operculum, postCG = postcentral gyrus, preCG = precentral gyrus, preCun = precuneus, PTC = parieto-temporal cortex, Put = putamen, R = right, RCT/P = parallel group randomized trial, RIO =  randomized intervention order, rpm = rotations per minute, SI = primary somatosensory area, SII = second somatosensory area, SMA = supplementary motor area, stimu = stimulation, STG = superior temporal gyrus, supCol = superior colliculi, Th = thalamus, TL = temporal lobe, Tpole = temporal pole, TTG = transverse temporal gyri, V1 = primary visual cortices, Y = yes.

#### Comparison of different needling depths

Of four studies, two studies [67], [68] found no significant difference between deep and superficial needling. Whereas Zhang et al. [25] found more activation in almost all brain areas from deep needling and Wu et al. [52] found more activation from superficial needling in the somatosensory area, motor area and language areas (Broca and Wernicke areas), and from deep needling more deactivation in the limbic system.

#### Comparison of electro-acupuncture vs. manual acupuncture

Overall, the results of three studies showed that electro-acupuncture tends to produce more activation and less deactivation compared to manual acupuncture. Regarding brain activations, two studies [58], [69] found more activation associated with electro-acupuncture in somatosensory areas, motor area, brainstem, cingulate or insula and one study [66] found no significant difference. Regarding brain deactivations, two studies [66], [69] showed manual acupuncture was associated with more deactivation in the limbic system [69], cuneus [66], transverse temporal gyrus [66] or middle frontal gyrus [66], yet two studies [58], [69] also showed more deactivation from electro-acupuncture in the septal area or precuneus.

#### Comparison of different frequencies of electro-acupuncture stimulation

Two studies compared different electro-acupuncture frequencies. Napadow et al. [58] found that the brainstem was more activated at 2 Hz than at 100 Hz. But Li et al. [66] found no significant difference between 2 Hz and 20 Hz.

#### Comparison of different intensities of manual acupuncture stimulation

Of six studies one study [70] observed that a longer duration of manipulation induced more activation in the inferior frontal, temporal, parietal gyrus, occipital lobe, cerebellum or temporal pole and more deactivation in the prefrontal cortex, orbital gyrus or pons than shorter manipulation. Four studies [42], [71]–[73] found more activation in the somatosensory areas, limbic system, visual, language areas or higher cognitive areas in response to stimulation compared to no stimulation. The last study [74] showed that stimulation which induced deqi by maximum manipulation was associated with more activation in the postcentral gyrus and the limbic system than stimulation that didn't induce deqi with minimum manipulation.

### Descriptive findings of differences between patients and healthy volunteers

All seven studies comparing healthy volunteers with patients showed that patients responded differently (See Table 2). According to Wang et al. [75] the frontal lobe was activated in stroke patients while motor areas were activated in healthy volunteers. Fu et al. [76] found patients with Alzheimer's disease had more activation in the cingulate gyrus and cerebellum. Liu et al. [77] found more robust activation in the hypothalamus in heroin addicts. Wu et al. [78] found deactivation in primary motor cortex (M1), parahippocampal gyrus, and higher cognitive areas and more activation in the cuneus and the insula in children with spastic cerebral palsy but not in healthy children. Conversely, more activation in caudate nucleus, thalamus and cerebellum was found in healthy children. Napadow et al. [79] compared patients with carpal tunnel syndrome (CTS) before and after five weeks' acupuncture to healthy volunteers receiving no treatment. Following acupuncture, a significant decrease in the activation area was found in contralateral primary somatosensory cortex (SI) and M1 in the CTS patients, as well as, increased separation between digit 3 and digit 2 cortical representations in SI, suggesting acupuncture-induced neuroplasticity. In addition, Napadow et al. compared manual acupuncture to cutaneous stimulation on both CTS patients and healthy volunteers. They found that CTS patients responded to verum acupuncture with less deactivation in the amygdala and greater activation in the lateral hypothalamic area [80], compared to healthy subjects. Moreover, CTS patients responded to sham acupuncture with greater activation in the somatosensory areas, cognitive and affective areas. Li et al. [17] found that stroke patients had more activation in the SI than healthy volunteers when both groups underwent both verum and sham acupuncture.

**Table 2:** Descriptive analysis of differences between patients and healthy volunteers.

Author (year)	Language	Stduyplace	Studydesign	Pat.NO.	HVNO.	Disease	Intervention	Control	Statistic	Response for both groups	Differences for both groups
Wang et al. 2004	C	CN	NCT	17	20	lesions in left central sulcus	EA (1 Hz, 0.1–03mA) ST36, GB34 (R)	N	Y	NA	Activation:1) Pat:Con. FL (the areas which are near the lesions and 3 cm anterior to central sulcus); 2) HV: SMA, MC
Fu et al. 2005	C	CN	NCT	6	6	Alzheimer's disease	EA (1 Hz) PC6 (R)	N	Y	Activation:Bil. TL, FL	Activation: Pat.>HV: CingG, Ce
Liu et al. 2007	E	CN	NCT	6	6	heroin addicts	MA ST36 (L)	N	Y	Activation:Con. Hyp, Th, paraHG	*Activation:1)Pat.>HV: Con. Hyp; 2)HV>Pat. Con. Th, paraHG*
Napadow et al. 2007*	E	US	NCT	13	12	Carpal tunnel syndrome	Electro-stimuli 100 Hz (Digit2, Digit3, Digit5) (Pat.: affected side; HV: dominant hand side)	N	Y	NA	1) Pat.: stimulating Digit3, decreased extent of activation: BA1, BA4; 2) no significant change in HV
Wu et al. 2008	E	CN	NCT	11	10	spastic cerebral palsy	MA (ERRM, rotating 2 Hz) LR3 (L)	N	Y	*Activation:Con. STG(BA22); Ipsi. H*	*1) Deactivation:Pat.>HV:Bil. MFG (BA10),preCG(BA4); Con. MTG(BA21), paraHG; Ipsi. SFG(BA8), IFG(BA46); 2) Activation:Pat.>HV: Bil. OL(Cun); Ipsi. IN ; HV>Pat.:Bil. Cau, Th, Ce*
Napadow et al. 2007	E	US	NCT	13	12	Carpal tunnel syndrome	MA (1.5 cm 1 Hz) LI4, (Pat.:affected side, HV:dominant hand side)	CS (1 Hz, monofilament) LI4 (Pat.:affected side, HV:dominant hand side)	Y	1)Activation:*Con.LHA*; 2)Deactivation:*Con Amyg;* pgACC, amACC, rspPCC, dlPFC, vmPFC, ant. IN, septal area, SI, SMA, Th	*1) Activation:Pat.>HV: LHA; 2) Deactivation:HV>Pat. :Amyg*
Li et al. 2006	E	CN	NCT	12	12	Stroke	EA (2 Hz) LI4,LI11 (L)	CS (1 Hz rough sponge brushing finger and palm) (L)	Y	Activation:CS>EA:Con. M1, SI	Activation: Pat.>HV: SI (for both CS and EA)

* published in Human Brain Mapping.

Words in italics means statistically significant;

amACC = anterior-middle anterior cingulate cortex, Amyg = Amygdala, BA = Brodmann area, Bil. = bilateral, C = Chinese, Cau = caudate nucleus, Ce = cerebellum, CingG = cingulate gyrus, CN = China, Con. = contralateral, CS = cutaneous stimulation, dlPFC = dorsolateral prefrontal cortex, EA =  electro-acupuncture, ERRM = even reinforcing and reducing method, FL = frontal lobe, H = hippocampus, HV = healthy volunteers, Hyp = hypothalamus, IFG = inferior frontal gyrus, IN = insula, Ipsi. = ipsilateral, L = left, LHA = lateral hypothalamic area, M1 = primary motor cortex, MA = manual acupuncture, MC = motor cortex, MFG = middle frontal gyrus, MTG = middle temporal gyrus, N = no, NA =  information unavailable, NCT =  non-randomized controlled trial, OL = occipital lobe, paraHG = parahippocampal gyrus, Pat. = patient, pgACC = pregenual cingulate cortex, preCG = precentral gyrus, R = right, rspPCC = retrosplenial posterior cingulate cortex, SI = primary somatosensory area, SFG = superior frontal gyrus, SMA = supplementary motor area, STG = superior temporal gyrus, Th = thalamus, TL = temporal lobe, vmPFC = ventromedial prefrontal cortex, Y = yes.

### Descriptive findings of differences between different acupuncture points

The data on acupuncture point specific changes in brain activation and deactivation are shown in Table S2, originating from 76 publications [15], [16], [22], [24], [27], [31], [33], [42], [45]–[47], [51]–[53], [55], [57]–[59], [61], [63], [64], [67], [69], [81]–[107] [29], [34], [37], [39], [40], [43], [73], [108]–[126] addressing 37 acupuncture points. Acupuncture points along the 12 regular meridians and one extra meridian (Du meridian) were assessed. The data showed changes in brain activity for each individual acupuncture point from respective publications. The most studied points were LI4, ST36, PC6, LR3 and GB34. These points have a wide clinical applicability and are frequently used in clinical practice. Overall the data showed that acupuncture stimulation mainly influenced the brain activity of the somatosensory areas, motor areas, auditory areas, visual areas, cerebellum, the limbic system and higher cognitive areas.

**Figure 3:**
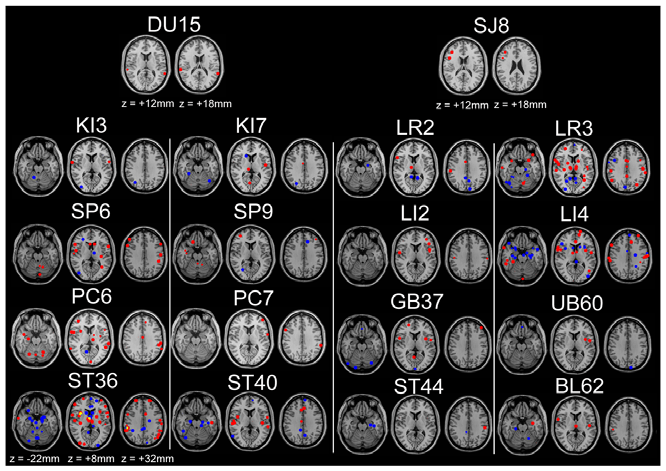
Map of brain response to 18 different acupuncture points. Red: activation; Blue: deactivation; Yellow: overlap.

Furthermore, we generated on a descriptive level map (Figure 3) of 18 acupuncture points from 46 publications, which reported pre-post data on Talairach coordinates. These 18 points were located along 9 meridians. The brain maps of each acupuncture point differ considerably from each other. However, the acupuncture points on the same meridian showed some similarities among the activation/deactivation pattern. For example, the points on the stomach meridian showed activation in the supramarginal gyrus and deactivation in the posterior cingulate, hippocampus, and parahippocampus. In addition, the vision related points GB37 and UB60 showed deactivation in the visual areas such as the cuneus.

### Descriptive findings of other comparisons and results

Besides our four main research questions, there are more research findings worth mentioning: comparisons between acupuncture and other stimulations; comparisons of acupuncture under different consciousness states; acupuncture at different time points; acupuncture at group of points; acupuncture effect correlated to expectation. Moreover, resting state functional connectivity was also investigated in several recently published papers.

#### Acupuncture vs. visual stimulation

Of four studies, Bai et al. [127] compared the stimulation phase and the resting phase of acupuncture stimulation and visual stimulation and found the BOLD signal returned to near-baseline values shortly after the visual stimulus, but for acupuncture stimulation the resting phase activities might be even higher than that of the stimulation phases. Hu et al. [71] and Gareus et al. [72] had contradictory results. Surprisingly, Hu et al. [71] reported no significant activation in the visual cortex during visual stimulation but from acupuncture stimulation, whereas Gareus et al. [72] found no activation in the visual cortex during acupuncture stimulation, and activation from visual stimulation. Li et al. [66] found both visual stimulation and acupuncture could activate the visual cortex.

#### Acupuncture vs. word generation paradigm

One study from Li et al. [29] found acupuncture at language specific acupuncture points SJ8 and Du15 did not activate the typical language areas in the left inferior frontal cortex which were activated during a word-generation task.

#### Acupuncture vs. finger tapping

Of three studies, both Kong et al. [39] and Hu et al. [81] found finger-tapping task can produce more reliable fMRI signal changes than that evoked by electro-acupuncture stimulation. However, Wang et al. [128] found no significant difference between electro-acupuncture at ST36, GB34 and a finger-tapping task.

#### Acupuncture in different states of consciousness (awake or anesthetized)

One study from Wang et al. [129] compared healthy subjects who underwent acupuncture at ST36 in two different consciousness states. The result showed activation in the awake state was greater than under anesthetic in the somatosensory area, the limbic system and basal ganglia.

#### Acupuncture at different time points

One study from Zeng et al. [130] compared acupuncture at KI3 and KI7 at two different time points – “open point time” and “closed point time” (the open or closed time point is determined by the Chinese medicine theory “Zi-Wu-Liu-Zhu”—the body's Qi and blood circulation schedule; acupuncture at the open point time results in maximum clinical effect and vice versa) [1]. The result showed acupuncture at “open point time” was associated with more deactivation in the frontal lobe, temporal lobe, cingulate cortex and cerebellum than acupuncture at the “closed point time”.

#### Acupuncture of group of points

In 29 papers [25], [35], [38], [44], [49], [62], [65], [68], [71], [74], [75], [93], [100], [109], [130]–[144] more than one acupuncture point was stimulated simultaneously. Of these groups of points, some were functional related, some were on the same meridian, some had close locations for electric stimulation, few were real acupuncture clinical formula. 15 of these 29 papers were included among our first four main questions. Overall the results of these studies were very heterogeneous and only three studies [93], [109], [136] reported an interaction effect between acupuncture points.

#### Acupuncture effect correlated to expectations

Three studies by Kong et al. [145]–[147] applied an expectancy model, and found positive expectation can increase acupuncture analgesia based on the objective fMRI signal changes in response to noxious stimuli. The study indicated that different mechanisms exist between acupuncture analgesia and expectancy evoked placebo analgesia. For the verum acupuncture group, there were only a few small differences (in primary motor cortex and middle frontal gyrus) between the high expectancy side and low expectancy side. However, for the sham acupuncture group, more differences were observed in contralateral operculum, ipsilateral insula, inferior frontal gyrus, medial frontal gyrus and superior frontal gyrus. So this result suggested expectancy might involve distinct mechanisms between verum acupuncture and sham acupuncture.

#### Functional connectivity modulated by acupuncture

Eight studies investigated functional connectivity of resting state. One of the first such studies (Dhond et al. study [57]) found that verum acupuncture, but not monofilament tapping increased resting state connectivity of the default mode network (DMN) to pain, affective and memory related regions of the brain. Verum acupuncture also increased sensorimotor network (SMN) connectivity to pain-related brain regions. Zhang et al. [148] and Bai et al. [149] found that acupuncture stimulation may induce the modulation of the “acupuncture-related” network, represented by significant changes of functional connectivity in several regions of the brain, such as the bilateral frontal gyrus, bilateral temporal gyrus, inferior parietal lobe, middle occipital gyrus, pre- and postcentral gyrus, anterior cingulate cortex (ACC), parahippocampus, insula, tonsil, pyramis, culmen, precuneus and cuneus. Qin et al. [150], [151] identified an amygdala-related network during the resting state both after verum and penetrating sham acupuncture at a nearby point. Compared to sham, verum acupuncture increased the connectivity between the amygdala, the PAG (periaqueductal gray) and the insula, and decreased the connectivity between the amygdala with the middle frontal cortex, the postcentral gyrus and the posterior cingulate cortex (PCC). Zhang et al. [119] compared the visual related functional networks between pre- and post- electro-acupuncture on the visual-related point GB37 and the non-visual related point KI8 and described a positive correlation between the pre-post resting states in visual networks for the GB37 group while an anti-correlation for the KI8 group. Liu et al. [152] found a similar result when comparing electro-acupuncture at GB37 and KI8. In addition, in a later study Liu et al. [153] reported that the DMN could be modulated after electro-acupuncture at the three acupuncture points (GB37, BL60 and KI8) and at a nearby sham point. As for intrinsic connectivity, the PCC and precuneus strongly interacted with other nodes during the pre- and post-stimulation states. The correlation was interrupted between the PCC/precuneus and the ACC. The orbital prefrontal cortex negatively interacted with the left medial temporal cortex only at the acupuncture points.

### Results from the ALE meta-analysis

A total of 34 studies were eligible for the inclusion criteria for the ALE meta-analyses (Table 3). A total of 10 meta-analyses were performed.

**Table 3:** Studies included in the ALE meta-analyses.

Author (year)	Intervention (verum)	Control (sham)	Subjects		Contrast		Included in following meta-analyses
			Intervention	Control	Pre-post	Between group	
Wang et al. 2007	EA(5 Hz random wave, 1–3mA) LI4 (R)	sham EA (5 Hz random wave, 1–3mA), NAP (1 cm apart from the right corner of the mouth) (R)	5	5	verum>rest; rest>verum; sham>rest; rest>sham		1a/b; 3a/b/c/d/e/f
Wang et al. 2006	MA (2.54 cm, ERRM, 1 Hz) LR3 (R)	sham MA, NAP (near LR3) (R)	10	10	verum>rest; sham>rest		1 a; 3a/c/e
Zhang et al. 2005	EA (3 cm, 2 Hz, 10 V), GB34, GB39 (L)	sham EA (3 cm, 2 Hz, 10 V), NAPs (3–4 cm lateral to GB34, GB39 respectively) (L)	16	18	verum>rest; rest>verum; sham>rest; rest>sham		1a/b; 3a/b/c/d/e/f
Hui et al. 2005	MA (2–3 cm, rotating 60 rpm) ST36 (R)	CS (tapping, monofilament), ST36 (R)	11	11	verum>rest; rest>verum; sham>rest; rest>sham		1a/b; 3a/b/c/d/e/f
Yoo et al. 2004	MA (1 cm, rotating, 2 Hz) PC6 (R)	1) sham MA (1 cm, rotating, 2 Hz), NAP (1.5–2 cm interior to PC6) (R); 2) CS (brushing, 2 Hz, monofilament) area unclear	12	12	verum>rest; sham>rest	verum>sham	1a; 2a; 3a/c/e
Wu et al. 1999	MA (1 cm, ERRM, 1–2 Hz) LI4 (L)	sham MA (5 mm, manipulation lightly), NAP (2–3 cm lateral fromST36) (L)	9	9	verum>rest; rest>verum; sham>rest		1a/b; 3a/b/c/e/f
Wu et al. 2002	EA (2–3 cm, 4 Hz) GB34 (L)	1) sham EA (2–3 cm, 4 Hz) NAP (4–5 cm lateral from GB34) (L); 2) mini EA (0.3–0.5 cm, 4 Hz, mini CUR), NAP (4–5 cm from sham point)	15	15	verum>rest; sham>rest	verum>sham	1a; 2a; 3a/c/e
Wang et al. 2009	EA (2 Hz, 0.8–1.8mA, continuous wave) ST42, ST36 (R)	sham EA (2 Hz, 0.8–1.8mA, continuous wave), NAP (at the depression inferior and posterior to the Capitula fibula), NAP (1 cun below GB 40)	30	10	verum>rest; rest>verum; sham>rest;		1a/b; 3a/b/c/e/f
Fang et al. 2008	MA (2–4 mm, rotating 160 rpm) LR3, LR2, ST44 (L)	sham MA(2–4 mm, rotating 160 rpm), NAP (metatarsal III and IV on the dorsum of the left foot) (L)	10	10	verum>rest; rest>verum; sham>rest; rest>sham		1a/b; 3a/b/c/d/e/f
Guan et al. 2008	EA (2 Hz, 10–20mA) GB37 (Bil)	sham EA (2 Hz, 10–20mA), NAP (Bil)	8	8	verum>rest; sham>rest		1a; 3a/c/e
Kong et al. 2007	EA (2 Hz) UB60, GB37 (R)	sham EA (2 Hz), NAP(1.5 cm post. and inf. to the small head of the fibula) (R)	6	6	verum>rest; rest>verum; sham>rest; rest>sham		1a/b; 3a/b/c/d/e/f
Fukunaga et al. 1999	EA (10–15 mm,4 Hz) LI4 (R)	CS (brushing, cosmetic brush 4 Hz) LI4 (R)	17	17	verum>rest; sham>rest		1a; 3a/c/e
Napadow et al. 2005	MA (ERRM, 1 Hz/2 Hz/100 Hz) ST36 (L)	CS (tapping, 1 Hz, monofilament) ST36 (L)	13	13	verum>rest; rest>verum; sham>rest;		1a/b; 3a/b/c/e/f
Chae et al. 2009	MA (0.8 cm, rotating 1 Hz) LR2 (L)	CS (unclear) LR2 (L)	10	10		verum>sham	2a
Chae et al. 2009	MA (0.8 cm, rotating 1 Hz) LR2 (L)	1) CS: covert (rotating 1 Hz) LR2 (L); 2) CS: overt (rotating 1 Hz) LR2 (L)	10	10		verum>sham	2a
Li et al. 2008	1) MA ST36 (R); 2) MA ST43 (R); 3) MA LR3 (R) 4) MA LR6 (R)	1) sham MA, NAP (dorsum between the first and second metatarsals, approximately 10 mm from the 2 real acupoints: ST43, LR3) (R); 2) sham MA, NAP (near ST36 and LR6) (R), same manipulation	1)9; 2) 9; 3)10; 4)8	1)7; 2)8		verum>sham	2a
Li et al. 2008	1) MA (15 mm, rotating,1 Hz) ST43 (R); 2) MA (15 mm, rotating,1 Hz) ST44 (R)	sham MA (15 mm, rotating,1 Hz) NAP (10 mm beside the two points)	1)9; 2)9	7		verum>sham	2a
Yan et al. 2005	1) MA (15 mm, ERRM, 1 Hz) LI4 (R); 2)) MA (15 mm, ERRM, 1 Hz) LR3 (R)	1)sham MA (15 mm, ERRM, 1 Hz), NAP1(10 mm anterior to LR3) (R); 2) sham MA (15 mm, ERRM, 1 Hz), NAP2(10 mm anterior to LI4) (R)	1)8; 2)10	1)7; 2)9		verum>sham; sham>verum	2a/b
Lu et al. 2008	MA (15 mm, ERRM, 1 Hz) LR6 (R)	sham MA (15 mm, ERRM, 1 Hz), NAP (lateral to LR6) (R)	8	8		verum>sham	2a
Dougherty et al. 2008	MA (ERRM, 180 rpm) LI4 (R)	Streitberger needle LI4 (R), manipulation gentlely	6	6		verum>sham; sham>verum	2a/b
Napadow et al. 2009	MA (1.5 cm, rotating, 0.5 Hz) PC6 (L)	CS (tapping, 0.5 Hz, monofilament) PC6 (L)	15	15		verum>sham	2a
Wang et al. 2005	MA (rotating, 2 Hz) BL62 (R)		6		verum>rest; rest>verum;		1a/b
Hou et al. 2002	MA (rotating, 2 Hz) LI4 (R)		6		verum>rest		1a
Kong et al. 2002	1)MA (rotating, 3 Hz) LI4 (L); 2) EA (3 Hz) LI4 (L)		11		verum>rest; rest>verum;		1a/b
Zhang et al. 2007	MA (25 mm) LI4, PC6, SP6, ST36 (R)		11		verum>rest; rest>verum;		1a/b
Li et al. 2005	MA (15 mm, ERRM, 1 Hz) LI4 (R)		6		verum>rest; rest>verum;		1a/b
MacPherson et al. 2008	MA (8–12 mm) LI4 (R)		17		verum>rest; rest>verum;		1a/b
Wu et al. 2007	MA (1.2cun) ST36 (R)		11		verum>rest; rest>verum;		1a/b
Chen et al. 2008	MA (0.3–0.5 cm, ERRM, 1–2 Hz) PC7 (R)		8		verum>rest		1a
Wang et al. 2007	EA (5 Hz, 1–3mA) LI4 (R)		6		verum>rest; rest>verum;		1a/b
Wu et al. 2008	MA (1.2cun) ST36, ST40 (R)		12		verum>rest		1a
Li et al. 2003	1) EA (2 Hz) SJ8 2) EA (2 Hz) DU15		18		verum>rest		1a
Deng et al. 2008	MA LI2 (non-dominant hand side)		13		verum>rest		1a
Li et al. 2006	MA (15 mm, ERRM, 1 Hz, 30 s/60 s/180 s) LI4 (R)		18		verum>rest; rest>verum;		1a/b

CS = cutaneous stimulation, CUR = current, EA = electro-acupuncture, ERRM = even reinforcing and reducing method, L = left, MA = manual acupuncture, NAP = non-acupuncture point, R = right.

**Figure 4:**
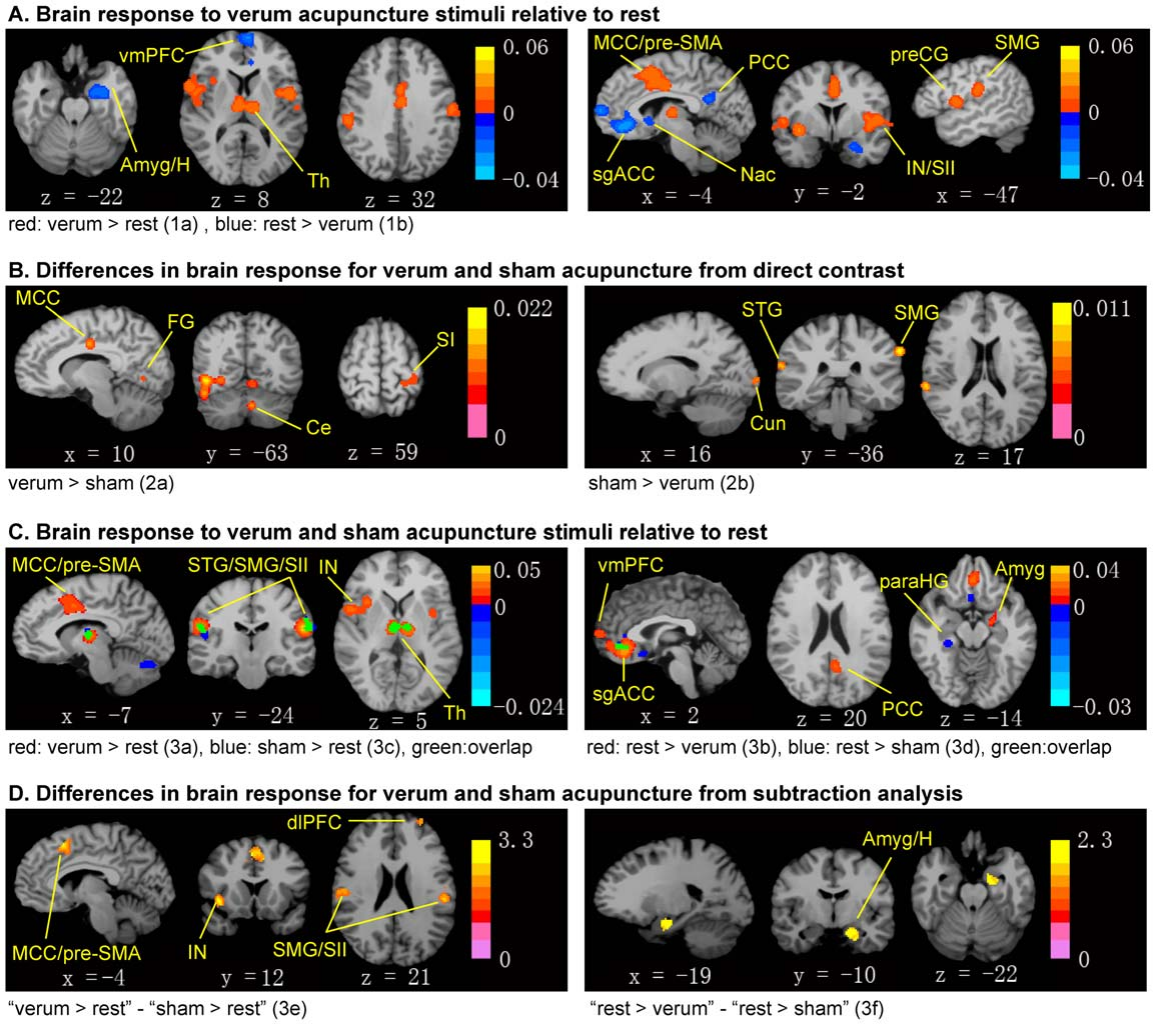
Results from the ALE meta-analyses. Meta-analyses were performed to evaluate brain response to acupuncture across studies, and contrast verum and sham acupuncture. (A) Brain response to verum acupuncture demonstrated activation in sensorimotor and affective/salience processing brain regions and deactivation in the amygdala and DMN brain regions. (B) Differences in brain response for verum and sham acupuncture from direct contrast showed significance in somatosensory areas, limbic regions, visual processing regions and cerebellum. (C) Brain response to verum and sham acupuncture individually demonstrated activation in sensorimotor and affective/salience processing brain regions and deactivation in the amygdala and DMN brain regions associated with verum acupuncture; while sham acupuncture produced activation in somatosensory regions, affective/salience processing regions, cerebellum and deactivation in limbic regions. (D) Differences in brain response between verum and sham acupuncture from subtraction analysis showed more activation in the sensorimotor affective/cognitive processing brain regions and more deactivation in the amygdala/hippocampal formation for verum acupuncture. For subfigures A–C, p<0.05, cluster level FDR corrected, color bar showed ALE value; for subfigure D, p<0.05, cluster level uncorrected, color bar showed Z value. Amyg: amygdala; Ce: cerebellum; dlPFC: dorsolateral prefrontal cortex; FG: fusiform gyrus; H: hippocampal formation; IN: insula; MCC: middle cingulate cortex; Nac: nucleus accumbens; paraHG: parahippocampal gyrus; PCC: posterior cingulate cortex; preCG: precentral gyrus; pre-SMA: pre-supplementary motor area; SI: primary somatosensory cortex; SII: secondary somatosensory cortex; sgACC: subgenual anterior cingulate cortex; SMG: supramarginal gyrus; Th: thalamus; vmPFC: ventromedial prefrontal cortex.

The meta-analysis for verum acupuncture stimuli on greater activation of verum acupuncture points compared to baseline (1a, verum>rest) included 36 experiments, 377 subjects and 470 foci. The result showed significant convergence in the supramarginal gyrus, secondary somatosensory cortex (SII), pre-supplementary motor area (pre-SMA), middle cigulate gyrus, insula, thalamus and precentral gyrus. The meta-analysis for greater deactivation of verum acupuncture points compared to baseline (1b, rest>verum) included 22 experiments, 219 subjects and 265 foci and the result revealed significant convergence in the subgenual anterior cingulate, subgenual cortex, amygdala/hippocampal formation, ventromedial prefrontal cortex (vmPFC), nucleus accumbens, and PCC (Table 4, Figure 4A).

**Table 4:** Clusters showing significant convergence for verum acupuncture points (FDR pN corrected at the cluster level, p<0.05) from ALE meta-analyses.

Brain region	BA	Talairach coordinates			ALE value	Volume (mm^3^)
		X	y	z		
**Verum>rest (1a)**						
Supramaginal gyrus/insula/SII	40	54	−26	24	0.0460	15440
	40	−54	−24	20	0.0565	8072
Pre-supplementary motor area/middle cingulate	6	−2	6	48	0.0318	9576
Thalamus		08	−16	8	0.0323	3776
Precentral gyrus	44	−46	−2	8	0.0259	3696
**Rest>verum (1b)**						
Anterior cingulate	32	0	34	−8	0.0406	6032
Subgenual cortex	25	2	8	−4	0.0202	1304
Amygdala/hippocampal formation		−28	−8	−24	0.0253	3240
Ventromedial prefrontal cortex	10	−2	60	10	0.0261	1728
Posterior cingulate	31	−6	−56	22	0.0188	1120

For the direct contrast of verum and sham acupuncture on greater activation from verum than sham acupuncture or greater deactivation for sham acupuncture (2a, verum>sham) we included in the meta-analysis 17 experiments, 156 subjects and 171 foci, resulting in significant convergence in fusiform gyrus, cerebellum, SI and middle cingulate gyrus. Whereas, on greater deactivation from verum than sham acupuncture or greater activation for sham (2b, sham>verum, 21 subjects, 3 experiments and 27 foci) the result showed significant convergence in supramarginal gyrus, superior temporal gyrus and cuneus (Table 5, Figure 4B).

**Table 5:** Clusters showing significant convergence for verum versus sham acupuncture (FDR pN corrected at the cluster level, p<0.05) from ALE meta-analyses.

Brain region	BA	Talairach coordinates			ALE value	Z value	Volume (mm^3^)
		x	y	z			
**Direct comparision verum>sham (2a)**							
Fusiform gyrus	37	44	−64	−6	0.0197		3720
Culmen of Vermis		−2	−66	−10	0.0160		1520
Cerebellar tonsil		−4	−58	−32	0.0224		1240
Postcentral gyrus	3	−20	−36	64	0.0116		992
Middle cingulate	24	10	−12	34	0.0165		904
**Direct comparision sham>verum (2b)**							
Supramarginal gyrus	40	−62	−34	34	0.0108		1120
Superior temporal gyrus	42	64	−34	20	0.0088		552
Cuneus	18	18	−98	0	0.0069		400
**Verum>rest (3a)**							
Middle cingulate/Pre-supplementary motor area	24	−2	2	38	0.0353		7392
Superior temporal gyrus	22	50	6	2	0.0262		6232
Supramarginal gyrus/SII	40	−54	−22	18	0.0484		6040
	40	56	−26	22	0.0402		4080
Thalamus		−8	−16	6	0.0326		4504
Insula	13	−38	−4	0	0.0177		2056
**Sham>rest (3c)**							
Tuber of vermis		0	−70	−24	0.0239		2664
Supramarginal gyrus/SII	40	−60	−22	22	0.0159		2424
Superior temporal gyrus	41	50	−32	16	0.0168		2320
	22	−52	10	−2	0.0180		808
Thalamus		6	−14	8	0.0206		1848
**Rest>verum (3b)**							
Anterior cingulate	32	0	32	−8	0.0413		5400
Amygdala/hippocampal formation	34	−18	−8	−20	0.0262		2632
Ventromedial prefrontal cortex	10	−2	60	10	0.0260		1784
Posterior cingulate	31	−6	−56	22	0.0193		1288
**Rest>sham (3d)**							
Anterior cingulate	32	−4	40	−2	0.0128		1720
Parahippocampal gyrus	36	28	−32	−14	0.0110		392
Subcallosal gyrus	25	4	14	−12	0.0108		360
**“Verum>rest” – “sham>rest” (3e)** *							
Pre-supplementary motor area/middle cingulate	6	4	12	46		2.7822	2120
Claustrum/insula		32	5	−1		3.2905	1848
Supramarginal gyrus/SII	40	−52	−26	22		2.4181	1728
	40	54	−18	24		2.2904	1168
Dorsolateral prefontral cortex	10	−28	57	23		2.2383	568
**“Rest>verum” – “rest>sham” (3f)** *							
Amygdala/hippocampal formation	34	−14	−9	−20		2.2768	1104

* uncorrected p<0.05.

The Subtraction analysis for verum versus sham acupuncture included in the first step analyses 3a–d for the pre-post contrast on verum or sham acupuncture compared to baseline (Table 5, Figure 4C). The analysis of greater activation of verum acupuncture than baseline (3a, verum>rest) included 234 subjects, 20 experiments and 305 foci and revealed significant convergence in middle cingulate gyrus, pre-SMA, superior temporal gyrus, supramarginal gyrus, SII, thalamus and insula. The analysis of greater deactivation of verum acupuncture compared to baseline (3b, rest>verum, 172 subjects, 15 experiments and 222 foci) came to the following significant convergence: subgenual anterior cingulate, amygdala/hippocampal formation, vmPFC and PCC. Comparing results on greater activation of sham acupuncture points than baseline (3c, sham>rest) from 164 subjects, 15 experiments and 200 foci, showed significant convergence in cerebellum, supramarginal gyrus, superior temporal gyrus and thalamus. Including data on greater deactivation of sham acupuncture points compared to baseline (3d, rest>sham) from 50 subjects, 5 experiments and 52 foci, resulted in significant convergence in pregenual anterior cingulate, subgenual cortex and parahippocampal gyrus.

Finally, in the contrast (subtraction) comparing the between-group differences for verum and sham acupuncture, significant differences between “verum>rest” and “sham>rest” (3e) as well as between “rest>verum” and “rest>sham” (3f) were identified. The subtraction analysis for “verum>rest” - “sham>rest” showed convergent activations in pre-SMA, middle cingulate gyrus, claustrum, insula, supramarginal gyrus, SII and dorsolateral prefrontal cortex (dlPFC). The subtraction analysis for “rest>verum” - “rest>sham” revealed convergence in amygdala/hippocampal formation (Table 5, Figure 4D).

## Discussion

Overall the results indicate that studies on acupuncture neuroimaging are very heterogeneous in terms of the study question, methodology and quality, this is the case in the descriptive analysis as well as in the meta-analysis.

From the descriptive view on the data it seems that compared to sham, verum acupuncture tended to be associated with more activation in the basal ganglia, brain stem, cerebellum, and insula and more deactivation was seen in the so-called “default mode network” and limbic brain areas, such as the amygdala and the hippocampus. In addition, a trend for more robust brain activation with greater intensity of acupuncture stimulation seems to be there. However, electro-acupuncture at low frequency also tended to activate a broader range of brain areas than electro-acupuncture at high frequencies. Furthermore, it looks like that patients responded to acupuncture stimulation with a more robust fMRI response compared to healthy volunteers. Acupuncture at different acupuncture points showed in the studies both similarities and differences between points. Finally, studies also suggested that acupuncture modulated the resting state connectivity within several noted networks including the default mode network, sensorimotor network, and amygdala-related network etc.

From the meta-analyses focusing only on brain response to verum acupuncture stimuli, activation was noted in supramarginal gyrus, SII, pre-SMA, middle cigulate gyrus, insula, thalamus and precentral gyrus, while deactivation was noted in pregenual anterior cingulate, subgenual cortex, amygdala/hippocampal formation, vmPFC, nucleus accumbens and PCC. Acupuncture specific effects were noted by meta-analyses of differences between verum and sham, which showed greater response in middle cingulate for verum compared to sham acupuncture. However, the results were variant within the different meta-analyses. The meta-analyses of direct contrast between verum and sham showed significant convergence for “verum>sham” in fusiform gyrus, cerebellum and SI, while for “sham>verum” in superior temporal gyrus, supramarginal gyrus and cuneus. Whereas, the subtraction meta-analyses of group-derived contrast showed greater activation from verum in pre-SMA, claustrum, insula, supramarginal gyrus, SII, dlPFC, greater deactivation from verum in amygdala/hippocampal formation. This heterogeneity suggests that group-derived contrast for verum and sham acupuncture tended to be above threshold in consistently specific brain areas, but were not significantly different in those areas, when assessed at the single study level.

### Strengths and limitations

To our knowledge this is the first systematic and extensive review on fMRI and acupuncture without any language restrictions. Besides the internationally well known databases such as Pubmed and EMBASE, less well known international databases such as the Chinese CNKI, the Japanese Ichushi WEB, and the Korean NDSL and KTKP were searched and the publications found were included in this review. Therefore, this very extensive review provides a transparent and detailed overview of the current literature available. In addition we structured the publications according to the research questions, such as the differences in brain activity associated with acupuncture stimuli between patients and healthy volunteers, to provide a good overview and a strong basis for future study designs, interventions, measurement methods, and possible diagnoses. Moreover, we complemented the systematic and comprehensive literature review with several ALE meta-analyses, providing analytic results for stronger evidence that are supported statistically. However, some studies reported direct contrast between verum and sham acupuncture groups, while some others reported pre-post contrast for each group, resulting in the fact that several meta-analyses had to be performed. The studies included in the descriptive review and the meta-analyses were highly heterogeneous regarding their study design, their aims and their quality of reporting. The reasons for these heterogeneous results are numerous, such as the varying acupuncture manipulation methods, different types of control arms, different methods of acquisition and analyzing the imaging data, the mainly investigated brain regions (region of interest) and the statistical analysis. The large variability between subjects and sessions with respect to the imaging data also needs to be taken into consideration [39], [154]. The imprecise nomenclature [155] is sometimes misleading, such as activation, deactivation, changes, baseline. We did not formally assess the quality of the publications, because no valid checklist for this type of research is available, though reporting guidelines are available and should be consulted by future research publications [156]. A narrative review including only studies that are considered to be of high quality would have overcome this problem. However the aim of this paper was to provide a systematic and broad overview for the first time using the publications currently available. We believe that many trials included in this review have limitations regarding their study design, analysis and reporting of their results. Hence, our results have to be interpreted with care. This is underlined by the multitude of contradictory results. Lastly, the field of research on brain imaging for acupuncture is evolving rapidly which may indeed lessen the relevance of older results using sub-optimal methodologies and analysis techniques.

### Discussion of results

The studies on BOLD activation and deactivation from a single point or a group of points came mainly from China and Korea. The controlled studies, including sham acupuncture as a control, were mainly from China and the US: the Chinese studies mainly used penetrating sham at a nearby non-acupuncture point as a control while the US studies mainly applied the non-penetrating Streitberger needle or monofilament tapping at the same acupuncture points. Studies on patients were mainly from China. Although we did not evaluate the quality of the publications, the papers published in English used a clearer reporting style than those published in other languages. The most innovative studies came from the US. These studies had clear study questions and explored acupuncture neurocorrelates with a pain matrix, expectation, autonomic regulation, somatosensory perception and deqi related brain response.

While in the descriptive analysis similarities were observed in the brain response to stimulation at different acupuncture points, some differences across points were also noted. For example, brain deactivation observed in the visual areas (precuneus, cuneus) appeared not only when the vision related points (GB37, UB60) were needled, but also when several non-vision related points (LR2, LR3, ST36) were needled, but not with the other points. One could argue, based on TCM theory, that for the two points on liver meridian (LR2, LR3), the liver opens into the eyes, reflecting its physiological and pathological conditions [157]. The stimulation of different acupoints in the same spinal segment could induce different fMRI activation patterns in the brain [142] while acupoints on the same meridian show some similarities in the activation/deactivation pattern [23].

The meta-analyses could only be done for publications that provided Talairach data, which was not the case for all of our study questions. The meta-analyses on the specific effect of acupuncture that compared verum and sham acupuncture came up with heterogeneous results. The subtraction analyses reflected descriptive results more than the direct contrast analyses. For example, subtraction meta-analyses confirmed more activation from verum in basal ganglia and insula, more deactivation in the limbic region of amygdala/hippocampal formation associated with verum, while meta-analyses of direct contrast for verum and sham confirmed more activation in cerebellum associated with verum. The convergence of brain regions shown for these meta-analyses comparing verum and sham acupuncture overlapped for middle cingulate gyrus. The first reason for the heterogeneous results might be the literature heterogeneity. Only two publications had both pre-post and between-group comparison results [15], [37]. Also, the different methods of acupuncture stimuli may have a strong impact of the result. Moreover, the direct contrast “verum>sham” included either more activation from verum or more deactivation from the sham. Thus, the results of direct contrast “verum>sham” and subtraction analysis “verum>rest” – “sham>rest” are not directly comparable. The ALE subtraction analysis for the comparison of verum versus sham acupuncture should be interpreted with caution because the groups are disparate in total number of foci. However, we refrained from randomly extracting experiments from the larger foci set [10], as this might have biased our results substantially. In particular, for the “rest>verum” – “rest>sham”, extracting 5 experiments out of 15 from “rest>verum” could most probably influence the result by chance. The meta-analysis of direct contrast for “sham>verum” included only three experiments and 27 foci. Hence this analysis might be with not enough power and doesn't represent the general. Nevertheless, we could see that brain regions such as SII, insula, cingulate gyrus, amygdala/hippocampal formation and prefrontal cortices might be important when differentiating the acupuncture specific effect from sham acupuncture. Acupuncture analgesia is considered as one of the most important indications for clinical acupuncture treatment [158], and those brain regions mentioned above are associated with the pain neuromatrix and might contribute in explaining the mechanism of acupuncture specific analgesia.

### Comparisons with other reviews

Some of the previous reviews [7], [8], [159]–[161] focused on a broader topic of neuroimaging techniques including EEG, PET, SPECT or MEG. Those reviews summarized research questions underlying certain acupuncture mechanisms, such as acupuncture analgesia, acupuncture placebo effect, specificity of meridian and acupuncture points, and acupuncture modulation on brain networks. They displayed the evidence for each research question and cited the relevant literature accordingly. However, in most cases the literature search was not transparently displayed. The other reviews [162]–[168] focusing on acupuncture and fMRI, had other emphases: Beissner et al. [162] focused on methodological problems, Cho et al. [163] explored neural substrates for hypothalamus-pituitary-adrenal axis and Chae et al. [164] reviewed traditional Korean acupuncture. The four Chinese narrative reviews on fMRI and acupuncture [165]–[168] discussed several research questions on the specific effects of acupuncture, such as different acupuncture points, manipulation methods, deqi or not deqi, and sham acupuncture. Our systematic literature review aimed to display the available studies as broad as possible and should offer a better and deeper overview on this topic, thus supporting future studies.

### Methodological consideration regarding future studies

One of the advantages for fMRI is that there are multiple possibilities by which experiments can be designed and data analyzed, providing information on different aspects of brain physiology. However, the inherent heterogeneity can complicate subsequent reviews and meta-analyses. Certain basic guidelines on proper statistical analyses of fMRI data should be followed, such as calculating difference maps if two conditions, such as brain response to stimulation at different acupoints, are to be contrasted. Furthermore, as suggested by Poldrack et al., publications relating to fMRI investigations of acupuncture should report all pertinent information relating to both imaging and acupuncture procedures [156]. Important topics include design and task specification, planned group comparisons, behavioral performance metrics, imaging details, data pre-processing, intersubject registration, statistical modeling details for both the individual and group level, and statistical inference including approach to multiple comparisons correction. Adoption of these guidelines will improve manuscript reviews and shorten the time to acceptance (or rejection), as well as facilitate the inclusion of publications in future reviews and meta-analyses.

### Conclusion

Brain response to acupuncture stimuli encompasses a broad network of regions consistent with not just somatosensory, but also affective and cognitive processing. While published results on acupuncture and fMRI were heterogeneous, from a descriptive perspective most studies suggest that acupuncture can modulate the brain activity within specific brain areas, and the evidence based on meta-analyses confirmed part of these results. Future studies should further improve methodological aspects and reporting related to both fMRI and acupuncture, and strictly control experimental conditions for more robust inference. Specifically, direct contrast analyses should be used to contrast different stimulus conditions (e.g. verum versus sham acupuncture) when evaluating research questions concerning acupuncture specificity.

## Supporting Information

Table S1 **Descriptive analysis of differences between verum and sham acupuncture.**
(DOCX)

Table S2 **Descriptive analysis of changes related to cortical and sub-cortical activation and deactivation at verum acupuncture points.**
(DOCX)
