# Supplementary material for: Characterizing Acupuncture Stimuli Using Brain Imaging with fMRI - A Systematic Review and Meta-Analysis of the Literature
Source: PLoS One. 2012 Apr 9;7(4):e32960. doi: 10.1371/journal.pone.0032960 (PMC3322129; doi:10.1371/journal.pone.0032960)
Supplement: Table S1 — Descriptive analysis of differences between verum and sham acupuncture. (DOCX) [file pone.0032960.s001.docx]

**Table S1: Descriptive analysis of differences between verum and sham acupuncture**

| **Author (year)** | **Language** | **Study place** | **Study design** | **Group NO.** | **Case NO.** | **Intervention** | **Control** | **Statistic** | **Group differences result in more activation** | **Group differences result in more deactivation** |
| --- | --- | --- | --- | --- | --- | --- | --- | --- | --- | --- |
| **a) Streitberger needle** | | | | | | | | | | |
| Yoo et al. 2007 | E | US | RIO, PB | 2 | 12 | MA (1cm, rotating, 2Hz) LI4 (R) | Streitberger needle LI4 ( R), same manipulation | Y | *verum>sham: BA3a, 4 and 6, PPL* | NA |
| Dougherty et al. 2008 | E | US | RCT/P, PB | 2 | 12 | MA (ERRM, 180rpm) LI4 (R) | Streitberger needle LI4 ( R), manipulation gentlely | Y | *1) verum>sham: Ipsi. MOFC (BA11, 25); 2) sham>verum: Bil. IPL (BA40), STG (BA22), P; Con. operculum, ant. IN* | verum>sham: Bil. BS; Con. IN |
| Deng et al. 2008 | E | US | RIO, PB | 2 | 20 | MA LI2 (non-dominant hand side) | Streitberger needle, NAP (ulnar side of the ipsi. forearm, 3 cm lat. to the PC6), same manipulation | Y | *verum>sham: Bil. IN, Fop, PO, RO (sham didin’t elicit any activation and deactivation in the ROIs)* | no deactivation detected |
| Schaechter et al.2007 | E | US | RCT/P | 2 | 8 | MA and EA (points unclear) | Streitberger needle (disconnected electro-stimu) | Y | *NSD in MC* | NA |
| **b) Acupuncture at non-acupuncture points in close proximity to acupuncture points** | | | | | | | | | | |
| Yoo et al. 2004 | E | US | RIO, PB | 2 | 12 | MA (1cm, rotating, 2Hz) PC6 (R) | sham MA (1cm, rotating, 2Hz), NAP (1.5-2cm interior to PC6) (R) | Y | *verum>sham: Con. preCG (BA4),SFG (BA10); Ipsi. Th, Ce* | NA |
| Li et al. 2008 | E | CN | RCT/P | 6 | 53 | 1) MA ST36 (R); 2) MA ST43 (R); 3) MA LR3 (R) 4) MA LR6 (R) | 1) sham MA, NAP (dorsum between the first and second metatarsals, approximately 10 mm from the 2 real acupoints: ST43, LR3) (R); 2) sham MA, NAP (near ST36 and LR6) (R), same manipulation | Y | *1) ST36>sham2: Ipsi.SPL (BA7), Ce; Bil. MOG (BA18),SI; Con. Opole (BA17), rACC; 2) ST43> sham1: Bil. SI, Ce; Ipsi. SII, IFG, Th; 3) LR3>sham1: Ipsi. MFG (BA10),MTG(BA21), rACC(BA24), LN, IN, SPL (BA7); Con. MOG(BA19); Bil. Th, Ce, SI; 4) LR6>sham2: Ipsi. SFG (BA9), MOG(BA19), dlPFC, SPL (BA7); Bil. LG, Ce, SI;* | NA |
| Xiao et al. 2008 | C | CN | RCT/P, PB | 2 | 26 | MA (3cm, RM), ST36 (R) | sham MA (3cm, RM), NAP (2cm lat. to ST36) (R) | Y | *verum>sham: TL* | NA |
| Zhang et al. 2007 | E | CN | NCT, PB | 2 | 12 | EA (2-3cm), GB34, GB39 (L) | sham EA (2-3cm), NAP(3-4cm ant. Lat. From G834. GB3) (L), same stimu | Y | *verum>sham: Bil. PFG (BA10, 11); Con. OCG (BA18)* | NA |
| Hu et al. 2006 | C | CN | RCT | 2 | 18 | 1) MA (deep needling) GB37, LR3; 2) MA (deep needling) ST40, ST43 | 1) sham MA (superficial needling), NAP1 (1 cun lat. to GB37), NAP2 (0.5 cun lat. to LR3); 2) sham MA (superficial needling), NAP1(2.5 cun lat. to ST40), NAP2 (1.5 cun lat. to ST43) | NA | verum>sham: latS, PAG, RN, PTC | NA |
| Wang et al. 2006 | C | CN | NCT | 2 | 18 | MA (2.54cm, ERRM, 1Hz) LR3 (R) | sham MA, NAP (near LR3) (R) | Y | *verum>sham: Bil. Ce, IFG, MFG, IPL, SPL, MTG, OL, paraHG, IN, Th, LN; Ipsi. postCG, TTG, IOG; Con. Tpole, Cun, ACC, PCC* | NA |
| Yan et al. 2005 | E | CN | RIO, PB | 4 | 37 | MA (15mm, ERRM, 1Hz) LI4, LR3 ( R) | sham MA (15mm, ERRM, 1Hz), NAP1(10mm anterior to LR3), NAP2(10mm anterior to LI4) (R) | Y | *1) LR3>NAP1: Bil. OL (BA19), Ce; Con. STG (BA42); Ipsi. MTG (BA21), post. Cing (BA29) 2) LI4> NAP2: Ipsi. MTG(BA21), Tpole(BA38), Ce (culmen)* | *1) LR3>NAP1: Bil. MFG (BA10); Con. IFG (BA44), IPL(BA40); Ipsi. SPL (BA7), STG (BA42), OL(BA17,18), ant. Cing (BA24), paraHG; 2) LI4> NAP2: Bil. MFG (BA10), IPL (BA40), Con. SFG (BA8), IFG (BA44), preCG (BA4), MTG (BA21), Th; Ipsi. STG (BA22)* |
| Li et al. 2003 | E | CN | NCT | 4 | 18 | 1) EA (2Hz) SJ8 2) EA (2Hz) DU15 | 1) sham EA (2Hz), NAP (1cm lat. SJ8) 2) sham EA (2Hz), NAP (1cm lat. DU15) | Y | 1) SJ8 >sham1: right IFG (BA44,46); 2) DU15>sham2: Bil. STG (BA22,42) (no significant activation with NAP) | NA |
| Lu et al. 2008 | C | CN | RCT/P, PB | 2 | 16 | MA (15mm, ERRM, 1Hz) LR6 ( R) | sham MA (15mm, ERRM, 1Hz), NAP (lat. to LR6) (R) | Y | verum>sham: Bil. Ce; Con. MEFG (BA6), OL (BA3), postCG (BA2), IPL (BA7), SPL (BA40,6), SFG (BA9,22), ITG (BA22); Ipsi. ACC (BA32,19,3), OL (BA18), SFG (BA8) | NA |
| Huang et al. 2009 | C | CN | RCT/P | 2 | 16 | MA (15±2mm, ERRM, rotating, 60rpm) SJ6 (R) | sham MA (15±2mm, ERRM, rotating, 60rpm), NAP(2cun above the dorsal wrist transverse striation, in the middle of Sanjiao and Small intestine meridian) (R) | Y | *ROIs activation points number: verum>sham: Ipsi. Ce* | NA |
| Choi et al. 2009 | K | KR | PB | 2 | 21 | MA (1cm) SP6 | Sham MA (1cm), NAP (3cm upper posterior to GB37) | Y | verum>sham: Hyp, Globus pallidus, SMG, Amgy; sham>verum: splenium of corpus callosum |  |
| Choe et al. 2002 | K | KR | PB | 2 | 5 | MA (2Hz) GB34 | NAP near GB34 | Y | verum>sham: Bil SPL (BA7), left M1 (BA4). ROI: somato-motor area |  |
| Li et al. 2008 | C | CN | RCT/P | 3 | 27 | 1) MA (15mm, rotating,1 Hz) ST43 (R); 2) MA (15mm, rotating, 1 Hz) ST44 (R) | sham MA (15mm, rotating,1 Hz), NAP (10mm beside the two points) | Y | *1) ST43>sham:Bil. SI/MI; Ipsi. Ce, SII, MEFG, IFG, ACC, Th; 2) ST44>sham:Bil. SI/MI, PCC; Ipsi. Ce, SII, LG, MOG; Con. MGF, IFG, preCun, LN* | NA |
| Lai et al. 2009 | C | CN | RCT/P | 2 | 16 | MA (15±2mm, ERRM, rotating, 60rpm) SJ5 (R) | MA (15±2mm, ERRM, rotating, 60rpm), NAP (2cun above the dorsal wrist transverse striation, in the middle of Sanjiao and Small intestine meridian) (R) | Y | *ROIs activation rate: verum>sham: Ipsi. Ce* | NA |
| Jeun et al. 2005 | E | KR | NCT | 2 | 12 | MA (rotating, 120rpm) GB34 (L) | sham MA (rotating, 120rpm), NAP (near GB34) (L) | Y | *verum>sham: Bil. SPL (BA7), Ipsi. M1 (BA4); sham>verum:Con. MFL (BA8)* | NA |
| Wu et al. 2002 | E | CN | RIO, PB | 2 | 15 | EA (2-3 cm, 4Hz) GB34 (L) | sham EA (2-3cm, 4Hz), NAP (4-5 cm lat. from GB34) (L) | Y | *verum>sham: Con. M1, SI, MEOC; Bil. PFC; Hyp* | *1) verum>sham: rACC; 2) sham>verum: Hyp* |
| Zhang et al. 2005 | C | CN | NCT | 2 | 20 | EA (3cm, 2Hz, 10V), GB34, GB39 (L) | sham EA (3cm, 2Hz, 10V), NAPs (3-4cm lat. to GB34, GB39 respectively) (L) | Y | sham>verum: Bil. ACC (BA24, 32); verum>sham: PAG, LN | 1) verum>sham: Bil. ACC(BA 24, 32); Ipsi. Amyg; Bil. H; 2) sham>verum: LN |
| Kong et al. 2007** | E | US | RIO | 3 | 8 | EA (2Hz) UB60, GB37 (R) | sham EA (2Hz), NAP (1.5 cm post. and inf. to the small head of the fibula) (R) | Y | *1) sham>UB60: SMA; 2)sham> GB37: Con. IN/operculum; SMA;* | NA |
| Kong et al. 2007* | E | US | RIO | 3 | 8 | EA (2Hz) UB60, GB37 (R) | sham EA (2Hz), NAP (1.5 cm post. and inferior to the small head of the fibula) (R) | Y | NA | NSD within OL |
| Fang et al. 2004 | E | CN | RIO, PB | 3 | 15 | MA (10mm, rotating 2Hz) LR3, GB 40 (L) | sham MA (10mm,ratating 2 Hz), NAP (dorsum of the foot, in the depression anterior to the junction of the third and fourth metatarsal) | Y | NSD | NA |
| Cho et. al. 2004 | K | KR | PB | 2 | 8 | MA (rotating 1Hz) LR3 | Sham MA (rotating 1Hz), NAP (2-3cm from LR3) | Y | NA | NSD of brain areas related to pain processing |
| Wang et al. 2009 | C | CN | NCT | 2 | 30 | EA (2Hz, 0.8-1.8mA, continuous wave) ST42, ST36 (R) | sham EA (2Hz, 0.8-1.8mA, continuous wave), NAP(at the depression inferior and posterior to the Capitula fibula), NAP(1 cun below GB 40) | Y | NA | NA |
| Wesolowski et al. 2009 | E | DE | RIO, PB | 2 | 20 | MA(0.5cm, rotating 3Hz) GB43 (L) | sham MA(0.5cm, rotating 3Hz), NAP(between the third and fourth toe proximal to the margin of the web) (L) | Y | *NSD in TTG* | NA |
| Fang et al. 2005 | C | CN | RIO | 4 | 10 | MA (2-4 mm, rotating 160rpm) LR3 ,LR2, ST44 (L) | sham MA( 2-4 mm, rotating 160rpm), NAP ( metatarsal III and IV on the dorsum of the left foot) (L) | Y | *1) LR3>sham: Bil. Th; 2) LR2>sham: Bil. Th, Con.Ipl; 3) ST44>sham: Con. Ipl; 4) sham>LR3: Con. STG, IFG; 5) sham>ST44: Con. IFG* | *1) LR3>sham: Con. MEFG, Ipsi. Cun, ACC; 2) LR2>sham: Con. MEFG, Ipsi. Cun; 3) ST44>sham: Ipsi. H, paraHG; 4) sham>LR3: Bil. post. CingG, Con. paraHG; 5)sham>LR2: Con. paraHG; 6) sham> ST44: Ipsi. Post. CingG, Con. paraHG;* |
| Fang et al. 2008 | E | CN | RIO | 4 | 10 | MA ( 2-4 mm, rotating 160rpm) LR3, LR2, ST44 (L) | sham MA( 2-4 mm, rotating 160rpm), NAP ( metatarsal III and IV on the dorsum of the left foot) (L) | Y | *1) LR2>sham: Ipsi. Tpole (BA38), Con. SMG (BA40); 2) no difference of LR3 and sham point* | *1) sham>LR2: Ipsi. Tpole (BA38); Bil. PCC (BA31); 2) sham>ST44: Ipsi. PCC (BA31);3) ST44>sham: Ipsi. RSC(BA30); 4) no difference of LR3 and sham point* |
| **c) Acupuncture at non-acupuncture points distant to acupuncture points** | | | | | | | | | | |
| Li et al. 2004 | E | CN | NCT | 10 | 67 | 1) EA GB34, GB35, GB39, ST36 (L); 2) EA LI4, LI11 (L); 3) EA LI9,LI14(L); 4) EA LI10, LI13 (L) 5) EA LI10, LI15(L) | 1) sham EA, NAPs near GB34, GB35, GB39, ST36 respectively (R); 2) sham EA, NAPs near LI4,LI11 respectively (R); 3) sham EA, NAPs near LI9,LI14 respectively (R); 4) sham EA. NAPs near LI10, LI13 respectively (R); 5) sham EA, NAPs near LI10, LI15 respectively (R) | Y | for sham: no significant change of cortex (no further details reported) | NA |
| Liu et al. 2004 | E | US | NCT | 2 | 7 | MA (1Hz) LI4 (R) | sham MA (1Hz), NAP(8–10cm post. to ST37) (R) | Y | verum>sham: PAG; BA3,40(Stimu of the NAP resulted in similar, but reduced frequency levels and lower activation volumes) | NA |
| Ai et al. 2004 | C | CN | NCT | 2 | 17 | MA (1cm) LR3 (R) | Sham MA (1cm), NAP(3cm lat. to ST36) (R) | Y | NA | NSD |
| Wang et al. 2007 | E | CN | RCT/P | 2 | 14 | EA (5Hz random wave, 1-3mA) LI4 (R) | sham EA (5 Hz random wave, 1-3 mA), NAP (1 cm apart from the right corner of the mouth) (R) | Y | NSD | *verum>sham: H, Amyg, CingG, FL* |
| Wu et al. 1999 | E | CN | Semi-RIO, PB | 2 | 9 | MA (1cm, ERRM, 1-2Hz) LI4 (L) | sham MA (5mm, manipulation lightly), NAP (2-3cm lat. fromST36) (L) | Y | *verum>sham: Con. Hyp, Nac, SI; Bil. PFC (BA8,9,10); sham> verum: Con. SMA, ACC (BA32), Fop (BA44, M1)* | *verum>sham: Bil. rACC (BA24b), H; Ipsi. OG, BG; Con. Amyg* |
| Napadow et al. 2009 | E | US | RIO, PB | 2 | 10 | EA(2-3cm,2-15Hz,dense-disperse wave, 2.10±0.96 mA) ST36 (randomized side) | sham EA (2-3cm, 2-15Hz,dense-disperse wave, 2.10±0.96 mA), NAP(8 cm above the proximal edge of the patella, on the midline of the thigh) (randomized side) | Y | *verum>sham: rostral vlPAG, SN, and right supCol* | *verum>sham: infCol; sham>verum: RN* |
| **d)** **Cutaneous stimulation at the same acupuncture points or sham site** | | | | | | | | | | |
| Hui et al. 2005 | E | US | NCT, PB | 2 | 15 | MA (2-3cm, rotating 60rpm) ST36 (R) | CS (tapping, monofilament) ST36 (R) | Y | sham>verum: SII | verum>sham: Amyg, H, paraHG, Hyp, Nac, Cing, IN, Tpole, Th, Cau, Put, PMC, SN, PN, Ce |
| Napadow et al. 2005 | E | US | NCT, PB | 2 | 13 | MA (ERRM, 1Hz ) ST36 (L) | CS (tapping, 1Hz, monofilament) ST36 (L) | Y | *verum>sham: Bil. ant. IN, post. IN, med. Middle Th, lat. Fpole; Con. dlPFC,IPL* | *verum>sham: Bil. H, vmPFC, dmPFC, Tpole; Ipsi. Amyg, Cing-subgenu, Fpole-dm* |
| Dhond et al. 2008 | E | US | RIO, PB | 2 | 15 | MA (1.5cm, 0.5 Hz) PC6 (L) | CS (tapping 0.5 Hz, monofilament) PC6 (L) | Y | Verum>sham: Bil. ACC; Con. Amyg, H, pre-SMA(BA8/6); Ipsi. PAG, SN, MTG(BA21), SMA(BA6), PPL(BA7), V1(BA17), Ce; sham>verum: TP (BA37/39) | sham>verum: Ipsi. MTG(BA21), ITG(BA20), dlPFC(BA8) |
| Hui et al. 2000 | E | US | NCT | 2 | 13 | MA (1cm, ERRM, rotating 120rpm) LI4 | CS (tapping, flexible wire, 120rpm) LI4 | Y | NSD | *verum>sham: Nac,Amyg, H,paraHG, Hyp,ventral tegmental area, ant.Cing (BA24), Cau, Put, ant. IN, Tpole* |
| Yoo et al. 2004 | E | US | RIO, PB | 2 | 12 | MA (1cm, rotating, 2Hz) PC6 (R) | CS (brushing, 2Hz, monofilament) area unclear | Y | *verum>sham: Con. preCG(BA4),SFG(BA10); Ipsi. Th, Ce* | NA |
| Yoo et al. 2007 | E | US | RIO, PB | 2 | 12 | MA (1cm, rotating, 2Hz) LI4 (R) | CS (brushing 2Hz, monofilament), dorsal aspect of the hand near LI4, (R) | Y | *verum>sham: Ipsi. IN; BA3a, 4 and 6, PPL* | NA |
| Huang et al. 2009 | C | CN | NCT | 2 | 8 | MA (15±2mm, ERRM, rotating, 60rpm) SJ5 (R) | CS(tapping, acupuncture needle, 180-200rpm) SJ5 (R) | Y | *ROIs activation intensity: verum>sham:Ipsi. Ce* | NA |
| Napadow et al. 2009 | E | US | RIO, PB | 2 | 15 | MA (1.5cm, rotating, 0.5Hz) PC6 (L) | CS (tapping, 0.5Hz, monofilament) PC6 (L) | Y | verum>sham: post.dmPFC, dlPFC; ant. dmPFC; sham>verum: Bil. SI (BA2), SII, LOC; Ipsi. Ant. IN | sham>verum: PCC, preCun, vmPFC, IPL, paraH, MTG/STG, Cun. |
| Chae et al. 2009 | E | KR | PB,RIO | 2 | 10 | MA (0.8cm, rotating 1Hz) LR2 (L) | CS (unclear) LR2 (L) | Y | *verum>sham: Ipsi. Cau, claustrum,,Cun(BA7); Bil. Post. Cing(BA29,31), fusiform gyrus(BA20,27); Con. MEFG(BA9) , preCun(BA31)* | *NA* |
| Wu et al. 2009 | C | CN | NCT | 2 | 8 | MA (15±2mm, ERRM, rotating, 60rpm) SJ5, PC6 (R) | CS (tapping, acupuncture needle, 180-200rpm) SJ5, PC6 (R) | Y | *ROIs activation intensity: verum>sham:Ipsi. PL* | NA |
| Hui et al. 2009 | E | US | PB,RIO | 4 | 48 | MA (rotating 1Hz) LI4, LR3, ST36 (R) | CS (tapping, monofilament) LI4, LR3, ST36 (R) | Y | *verum >sham: Th, Ipsi. ant. IN, Cing-am, post. Cing (BA23d); sham >verum: Con. SII* | *verum>sham:Fpole, Cing,Hyp* |
| Schockertet al. 2009 | G | DE | NCT | 4 | 36 | MA (Yamamoto new scalp acupuncture) point C, basal ganglia, cerebellum | CS (fingernail pressure), NAP (middle point of SJ23 and GB14) | Y | verum>sham: SMC,PMC | NA |
| Fukunaga et al. 1999 | J | JN | RIO | 2 | 17 | EA (10-15mm,4Hz) LI4 (R) | CS (brushing, cosmetic brush 4Hz) LI4 (R) | Y | verum>sham: Bil. MEFG, CingG; Con. IN; sham>verum: Con. postCG; | NA |
| Zou et el. 2008 | C | CN | NCT | 2 | 8 | MA (15±2mm, ERRM, rotating, 60rpm) SJ5, SJ6 (R) | CS (tapping, acupuncture needle, 180-200rpm) SJ5, SJ6 (R) | Y | *NSD* | NA |
| Huang et al. 2009 | C | CN | NCT | 2 | 7 | MA (15±2mm, ERRM, rotating, 60rpm) PC6 (R) | CS (tapping, acupuncture needle, 180-200rpm) PC6 (R) | Y | *NSD* | NA |
| Huang et al. 2009 | C | CN | NCT | 2 | 8 | MA (15±2mm, ERRM, rotating, 60rpm) SJ6 (R) | CS (tapping, acupuncture needle, 180-200rpm) SJ6 (R) | Y | *NSD* | NA |
| Chae et al. 2009 | E | KR | RIO | 3 | 10 | MA (0.8cm, rotating 1Hz) LR2 (L) | 1) CS: covert (rotating 1Hz) LR2 (L); 2) CS: overt (rotating 1Hz) LR2 (L) | Y | *1) verum>covert: Ipsi. MFG(BA9), Put; Con. preCun(BA7), M1(BA4); 2) verum>overt: Bil. ant. CingG(BA24,32); Con. M1(BA4), preCun(BA7), STG(BA38), ITG(BA20), SFG(BA8), IN* | NA |
| Li et al. 2006 | E | CN | NCT | 2 | 24 | EA (2Hz) LI4, LI11 (L) | CS (1Hz rough sponge brushing finger and palm) (L) | Y | *1)HV: CS>EA: Bil. M1, SI, SII, Ce; 2) Pat. CS>EA: Con. M1, SI;* | NA |

*published in Human Brain Mapping

**published in NeuroImage

Words in italics means statistically significant;

ACC=anterior cingulate cortex, Amyg=Amygdala, ant.=anterior, BA=Brodmann area, BG=basal gyrus, Bil.=bilateral, BS=brainstem, C=Chinese, cACC=caudal anterior cingulate cortex, Cau=caudate nucleus, Ce=cerebellum, Cing=cingulate, Cing-am=anterior middle cingulate, CingG=cingulate gyrus, Cing-subgenu=Cingulate subgenual, Con.=contralateral, CS=cutaneous stimulation, Cun=cuneus, DE=Germany, dlPFC=dorsolateral prefrontal cortex, dmPFC=dorsomedial prefrontal cortex, E=English, EA=electro-acupuncture, ERRM=even reinforcing and reducing method, FL=frontal lobe, Fop=frontal operculum, Fpole-dm= dorsomedial frontal pole, G=German, H=hippocampus, HV=healthy volunteers, Hyp=hypothalamus, IFG=inferior frontal gyrus, IN=insula, inf.=inferior, infCol=inferior colliculi, IOG=inferior occipital gyrus, IPL=inferior parietal lobule, Ipsi.=ipsilateral, ITG=inferior temporal gyrus, J=Japanese, JN=Japan, K=Korean, KR=Korea, L=left side, lat.=lateral, latS=lateral sulcus, LG=lingual gyrus, LN=lenticular nucleus, M1=primary motor cortex, MA=manual acupuncture, MC=motor cortex, med.=medial, MEFG=medial frontal gyrus, MEOC=medial occipital cortex, MFG=middle frontal gyrus, MFL=middle frontal lobe, MOFC=medial orbitofrontal cortex, MOG=middle occipital gyrus, MTG=middle temporal gyrus, NA=information unavailable, Nac=nucleus accumbens, NAP=non-acupuncture point, NCT=non-randomized controlled trial, NSD=non statistically different, OCG=occipital gyrus, OG=orbital gyrus, OL=occipital lobe, Opole=occipital pole, P=pons, PAG=periaqueductal gray, paraH=parahippocampus, paraHG=parahippocampal gyrus, Pat.=patient, PB=patient blinded, PCC=posterior cingulate cortex, PFC=prefrontal cortices, PFG=prefrontal gyrus, PL=parietal lobule, PMC=premotor cortex, PN=pontine nuclei, PO=parietal operculum, post.=posterior, postCG=postcentral gyrus, PPL=posterior parietal lobe, preCG=precentral gyrus, preCun=precuneus, PTC=parieto-temporal cortex, Put=putamen, R=right side, rACC=rostral anterior cingulate cortex, RCT/P=parallel group randomized trial, RIO=randomized intervention order, RN=red nucleus, RO=rolandic operculum, ROI=region of interest, rpm=rotations per minute, RSC=retrosplenial cortex, SFG=superior frontal gyrus, SI=primary somatosensory area, SII=second somatosensory area, SMA=supplementary motor area, SMC=supplementary motor cortex, SMG=supramarginal gyrus, SN=substantia nigra, SPL=superior parietal lobule, stimu=stimulation, STG=superior temporal gyrus, supCol=superior colliculi, Fpole=frontal pole, Th=thalamus, TL=temporal lobe, TP=temporo-occipital cortex, Tpole=temporal pole, TTG=transverse temporal gyri, V1=primary visual cortices, vl-=ventrolateral, vmPFC=ventromedial prefrontal cortex, Y=yes.
