# Supplementary material for: Characterizing Acupuncture Stimuli Using Brain Imaging with fMRI - A Systematic Review and Meta-Analysis of the Literature
Source: PLoS One. 2012 Apr 9;7(4):e32960. doi: 10.1371/journal.pone.0032960 (PMC3322129; doi:10.1371/journal.pone.0032960)
Supplement: Table S2 — Descriptive analysis of changes related to cortical and sub-cortical activation and deactivation at verum acupuncture points. (DOCX) [file pone.0032960.s002.docx]

Table S2: Descriptive analysis of changes related to cortical and sub-cortical activation and deactivation at verum acupuncture points

| **Points (body side)** | **Brain activation areas** | **Brain deactivation areas** |
| --- | --- | --- |
| **LI4 (R)** | **Bil.** SII[[16]](#_ENREF_10), preCG[94](BA4)[95], postCG[94], H[96], Amyg[96], IN[16,94], ros. CingG[96], Hyp[[52]](#_ENREF_44), STG[94,96], TL[96], MFG[94], MTG[94], Tpole[[47]](#_ENREF_39), PFC(BA8,9,10)[[52]](#_ENREF_44), PO(BA40)[[52]](#_ENREF_44) PMA[96]; **Con.** preCG[47,96,97](BA4)[[69]](#_ENREF_61), postCG(BA1,2,3)[95,97](BA3,2,40)[[69]](#_ENREF_61), SI[16,52], IN(BA13)[97], CingG[109](BA24,32)[97], Amyg[97], Th[95], NAC[[52]](#_ENREF_44), LN[95], PAG[95], SPL[96], Ce[47,95,96], inf. Semi-lunar lobe of Ce[98],LG[94], IPL[94], SMA[[52]](#_ENREF_44), SMG[[47]](#_ENREF_39), STG[[47]](#_ENREF_39), occipitotemporal junctional zone(BA37)[97], MTG(BA37)[97], MEFG(BA6)[95,97], MFG[109]; **Ipsi.** postCG(BA43,2)[97], preCG(BA6)[97], Put[[69]](#_ENREF_61), SFG[109](BA9)[99],MFG[109](BA8)[95], IFG(BA46)[95], preCun[47,94,96], O[94], STG(BA22)[97](BA38)[95], IPL(BA40)[97], inf. Semi-lunar lobe of Ce[97,109], posterior lobe of cerebellum declive[97], sup. Semi-lunar lobe of Ce[98], MTG(BA21,20)[97], MEFG(BA8)[97],ITG(BA37)[97], PFC(BA6)[[52]](#_ENREF_44); **Unclear side**: SI[[59]](#_ENREF_51), SII[[59]](#_ENREF_51), post. Cau ACC(BA24)[[52]](#_ENREF_44), Th[[47]](#_ENREF_39), Ce[[67]](#_ENREF_59), FL[100], OL[100] | **Bil.** preCG(BA4, 6)[97], CingG[47,96], ant. CingG(BA32,24)[95], paraHG[47,96], H[47,52], Amyg[[47]](#_ENREF_39), AG[96], PFC[96], PFL[[47]](#_ENREF_39), OL[47,96], MEFG(BA9)[95], Cau[95], Cun(BA18)[95], STG(BA22)[[69]](#_ENREF_61); **Con.** Amyg[[52]](#_ENREF_44), IFG[47,109], MFG[109](BA46)[95], STG(BA38,22)[95], ITG(BA20)[95], LG(BA18)[95], MTG[109](BA21)[97](BA20)[95], IOG(BA19)[97], MOG(BA19)[97], preCun[109](BA7)[[69]](#_ENREF_61), LN[[69]](#_ENREF_61), postCG[109]; **Ipsi.** ros. ACC(BA24b)[[52]](#_ENREF_44), post. Cing(BA30)[[69]](#_ENREF_61), SPL[96], MEFG(BA11)[97], SFG(BA9)[97], FG(BA37,19)[95], IOG(BA18,19)[95], MFG[109], IFG[109], preCG[109]; **Unclear side**: ant. IN[[59]](#_ENREF_51), Nac[[59]](#_ENREF_51), Amyg[[59]](#_ENREF_51), H[[59]](#_ENREF_51), paraH[[59]](#_ENREF_51), Hyp[[59]](#_ENREF_51),ventral tegmental area[[59]](#_ENREF_51), ant. CingG[[59]](#_ENREF_51), OL(BA19)[[67]](#_ENREF_59), FL,MEFG(BA10)[[67]](#_ENREF_59), Cau[[59]](#_ENREF_51), Put[[59]](#_ENREF_51), Tpole[[59]](#_ENREF_51) |
| **ST36 (Bil)** | **Bil.** OL[101], MTG[101]; **Left** postCG[101], post. Ce[101], preCun[101], IPL[101]; **right** post. and ant.CingG[101] |  |
| **ST36 (Uni)** | **Bil.** SII[[53]](#_ENREF_45), postCG[102], IN[58,94], Cing-am[[58]](#_ENREF_50), Th[[58]](#_ENREF_50), TL[24,99,102], MTG[94,103], SFG[94], MFG[94], PL[[53]](#_ENREF_45), Dors. NRP[[53]](#_ENREF_45), PFC(BA8,9,10)[[52]](#_ENREF_44), lat. Fpole[[58]](#_ENREF_50), dl. PFC[[58]](#_ENREF_50), IPL[[58]](#_ENREF_50), dmPFC[[58]](#_ENREF_50); **Con.** preCG[94], postCG[94], SI[52,58], H(BA36)[111,112], Hyp[52,112], NAC[[52]](#_ENREF_44), MTG(BA41)[111], O[94], AG[94], IN(BA13)[104], Put[[58]](#_ENREF_50), NRP[[58]](#_ENREF_50), sub-gyral of FL[104], PFL[103]; **Ipsi.** preCG[104], CingG(BA24)[104], Hyp[99,102], Cing-pm[[58]](#_ENREF_50), Th[[58]](#_ENREF_50), STG (BA22)[103,111], ITG[103], BS[103], SMG(BA40)[111], SOG[94], IPL[104], IFG[[105]](#_ENREF_97), PVN[99]; inter-hermispheric(partA)[104], FOC[[58]](#_ENREF_50), PO(BA40)[[52]](#_ENREF_44); **Unclear side:** Hyp**[106]**, FL[106], P[106], postCG[[105]](#_ENREF_97)(BA2)[[107]](#_ENREF_99); rostral vlPAG[[51]](#_ENREF_43), SN[[51]](#_ENREF_43), supCol[[51]](#_ENREF_43), MFG(BA3, 6)[[107]](#_ENREF_99), IN(BA13)[[107]](#_ENREF_99), preCun(BA19)[[107]](#_ENREF_99),STG(BA 21, 22)[[107]](#_ENREF_99), MTG(BA38, 39)[[107]](#_ENREF_99), IPL(BA40)[[107]](#_ENREF_99); | **Bil.** Amyg[[53]](#_ENREF_45), Hyp[[53]](#_ENREF_45), septal area[[53]](#_ENREF_45), NAC[[53]](#_ENREF_45), ant. Cing-subgenual[[53]](#_ENREF_45), pgACC[[53]](#_ENREF_45), Cing-pm[[53]](#_ENREF_45), CingG[[105]](#_ENREF_97), PCC[[53]](#_ENREF_45), ros. ACC(BA24b)[[52]](#_ENREF_44), retrosplenial cortex[[53]](#_ENREF_45),paraHG[53,105], H[52,53,58,105], preCun[[105]](#_ENREF_97), Ce[[105]](#_ENREF_97), Tpole[53,58], Fpole[[53]](#_ENREF_45), vmPFC(BA11,12,25)[53,58], Cau[[53]](#_ENREF_45), post. Th[[53]](#_ENREF_45), IPL[[53]](#_ENREF_45), PMC[[53]](#_ENREF_45), SN[[53]](#_ENREF_45), reticular formation[[53]](#_ENREF_45), Pontine Nuclei[[53]](#_ENREF_45) , PFC[[58]](#_ENREF_50), FOC(EA100Hz)[[58]](#_ENREF_50) ;**Con.** ant. CingG[104], Amyg[52,58], Cing-subgenu[[58]](#_ENREF_50), dm. Fpole[[58]](#_ENREF_50), post. Cing[[58]](#_ENREF_50);**Ipsi**. H[[58]](#_ENREF_50), septal area[[58]](#_ENREF_50), LN[104], orbital and basal gyri[[52]](#_ENREF_44), vmFpole[[58]](#_ENREF_50); **Unclear side**: inter-hemispheric(partB)[104], NRP[51], NTS/VNC[51], PBN[51], LC[51], RtTg/PN,[51] NCF[51], infCol[51], caudal vlPAG[51], lat. PAG[51] |
| **PC6 (Uni)** | **Bil.** SI[[55]](#_ENREF_47), SII[[55]](#_ENREF_47) (BA40,43)[[15]](#_ENREF_9), preCG[55,94], postCG[94], AG[[55]](#_ENREF_47), SMG[[55]](#_ENREF_47), dmPFC[[55]](#_ENREF_47), ACC(BA24,32)[[57]](#_ENREF_49), FL[63], PL[63], TL[63], STG[81,94](BA22,38)[[15]](#_ENREF_9), MTG[82,94](BA37)[[15]](#_ENREF_9), SFG[82], IFG[82], MFG[94], CingG[82,94], Ce[[15]](#_ENREF_9)(pars anterior lobuli quadrangularis) [82], IN[[15]](#_ENREF_9), TTG(BA42)[[15]](#_ENREF_9);**Con**. Amyg[[57]](#_ENREF_49), H[[57]](#_ENREF_49), preSMA(BA8/6)[[57]](#_ENREF_49), O[94], IN[94] (BA13)[81], Hyp[94], SMG[94], IPL[94](BA40)[[15]](#_ENREF_9), preCG(BA4)[81], postCG(BA2,3)[81](BA3,1,2)[[15]](#_ENREF_9), OL[63], TTG[81], MEFG(BA8)[[15]](#_ENREF_9), Th[[15]](#_ENREF_9); Ipsi. STG[82], PAG[[57]](#_ENREF_49), SN[[57]](#_ENREF_49), MTG(BA21)[[57]](#_ENREF_49), SMA(BA6)[[57]](#_ENREF_49), post. PC(BA7)[[57]](#_ENREF_49), V1(BA17)[[57]](#_ENREF_49), Ce[57,81];[[15]](#_ENREF_9) | **Con.** central sulcus[[55]](#_ENREF_47); **Unclear side:** preCun[[55]](#_ENREF_47), PCC[[55]](#_ENREF_47), IPL[[55]](#_ENREF_47), MEOG[[55]](#_ENREF_47), dlPFC(BA9/10/44/45/46)[[55]](#_ENREF_47); |
| **LR3 (Bil)** | **Unclear side:** BA4(preCG, MFG)[[83]](#_ENREF_75), BA13[[83]](#_ENREF_75), BA18[[83]](#_ENREF_75), BA19[[83]](#_ENREF_75), BA22 (STG, IN, preCG)[[83]](#_ENREF_75), BA31[[83]](#_ENREF_75), BA39[[83]](#_ENREF_75), BA44 (preCG)[[83]](#_ENREF_75) | **Unclear side:** BA4 (preCG)[[83]](#_ENREF_75), BA6[[83]](#_ENREF_75), BA9 (MFG)[[83]](#_ENREF_75), BA19[[83]](#_ENREF_75), BA36[[83]](#_ENREF_75), BA37 (FG)[[83]](#_ENREF_75), BA39[[83]](#_ENREF_75) |
| **LR3 (Uni)** | **Bil.** amACC(BA24,32)[[46]](#_ENREF_38), SMC(BA6)[[46]](#_ENREF_38), Th[27,46], IN[27,42,46], SII[[42]](#_ENREF_34)(BA40,43)[[46]](#_ENREF_38), Ce[27], SFG(BA6,8)[27], MFG(BA46)[27], SPL(BA7)[27], IPL(BA40)[27], paraHG[27], LN[27], MEOG(BA19)[27], postCG[45]; **Con.** Tpole(BA38)[27], Cun(BA19)[27], ant. lobe of Ce[98], STG[45], Th[42,45], BAGA[[42]](#_ENREF_34), MFG[109]; **Ipsi**. preCG[45], IPL[45], SI(BA3,1,2)[[46]](#_ENREF_38), Ce[84], SFG[84], LG(BA18,19)[84], PCC(BA30)[84], postCG(BA1)[27], MTG(BA21)[27], TTG(BA42)[27], IOG(BA18)[27], inf. Semi-lunar lobe of the posterior lobe of Ce, post. ITG[[42]](#_ENREF_34), orbital IFG[[42]](#_ENREF_34); **Unclear side**: post CingG[27], ant. CingG[27], TL[100], BA2,3,43(postCG)[[83]](#_ENREF_75), BA6(preCG, SFG)[[83]](#_ENREF_75), BA9(IFG)[[83]](#_ENREF_75), BA10(MFG)[83], BA22(STG)[83], BA40(IPL)[83], BA43(PostCG)[83]; | **Bil.** Fpole(BA10)[[46]](#_ENREF_38), H[[46]](#_ENREF_38), paraH[[46]](#_ENREF_38), Tpole(BA38)[[46]](#_ENREF_38), preCun(BA7)[[46]](#_ENREF_38), PCC(BA31)[[46]](#_ENREF_38), rspPCC(BA29,30)[[46]](#_ENREF_38); **Con.** Cun(BA18,19)[[46]](#_ENREF_38), SFG[109], CingG[109]; **Ipsi.** MFG[109]; **Unclear side:** BA6(preCG)[[83]](#_ENREF_75), BA18(Cun, MOG, LG)[[83]](#_ENREF_75), BA19(FG,MOG,LG)[83], BA28,35(paraHG)[83], BA30(post. Cing)[83], BA31 (post. Cing, preCun)[83], BA37[83]; |
| **LI2 (Uni)** | **Bil.** PO[[22]](#_ENREF_16), RO[[22]](#_ENREF_16), Fop[[22]](#_ENREF_16), IN[[22]](#_ENREF_16) |  |
| **LI11 (R)** | **Bil.** SI[85], SII[85], SPL[85], IPL[85], MTG[85], STG[85], preCun[85]; **Con.** CingG[85], SN[85], Hyp[85], Aq[85], H[85], Cau[85]; **Ipsi.** M1[85], SMA[85]; |  |
| **SP6 (Bil)** | **Unclear side:** BA3[[86]](#_ENREF_78), BA7[[86]](#_ENREF_78), BA13(IN)[[86]](#_ENREF_78) | **Unclear side:** BA6(preCG)[[86]](#_ENREF_78), BA38(STG)[[86]](#_ENREF_78), BA47[[86]](#_ENREF_78) |
| **SP6 (Uni)** | **Bil.** MTG[94], IFG[94], MEFG[110], paraCL[110], preCG[110], postCG[110], IN[110], Th[110], STG[110], TTG[110], preCun[110]; **Con.** preCG[94], postCG[94], IPL[94,110], IFG[110]; **Ipsi.** SPL[94], IN[94], MFG[110], IFG[[105]](#_ENREF_97), MTG[110], CingG[110]; **Unclear side:** postCG[[105]](#_ENREF_97), Ce[[87]](#_ENREF_79), BAGA[[87]](#_ENREF_79); BA5[[86]](#_ENREF_78), BA6[[86]](#_ENREF_78), BA13(IN)[[86]](#_ENREF_78), BA17(LG)[[86]](#_ENREF_78), BA18,19,31(CingG)[[86]](#_ENREF_78), BA38(STG)[[86]](#_ENREF_78), BA40(IPL)[[86]](#_ENREF_78) | **Bil.** paraHG[[105]](#_ENREF_97), H[[105]](#_ENREF_97), CingG[[105]](#_ENREF_97), preCun[[105]](#_ENREF_97), Ce[[105]](#_ENREF_97); **Unclear side**: BA3(postCG), BA4(preCG)[[86]](#_ENREF_78), BA18(IOG)[[86]](#_ENREF_78), BA21(MTG)[[86]](#_ENREF_78), BA36[[86]](#_ENREF_78), BA38(STG)[[86]](#_ENREF_78), BA39(MTG)[[86]](#_ENREF_78) |
| **SP9 (R)** | **Bil.** STG[96]; Ipsi. H[96], PFC[96], SII[96]; **Unclear side:** BA13(IN)[[88]](#_ENREF_80), BA19(paraHG)[[88]](#_ENREF_80), BA22(STG)[[88]](#_ENREF_80), BA39(MTG)[[88]](#_ENREF_80), BA40(IPL)[[88]](#_ENREF_80), BA47[[88]](#_ENREF_80) | **Bil.** ITL[96], PFC[96]; **Ipsi**. preCun[96] |
| **ST32 (L)** | **Ipsi.** FL[112]; **Unclear side:** Hyp[106], FL[106], P[106] |  |
| **ST40 (R)** | **Con.** STG(BA22,39)[111], MTG(BA21)[111], STG(BA22,41)[111]; **Ipsi**. CingG(BA24)[111], MEFG(BA6)[111], paraCL(BA6)[111]; **Unclear side**: IN[89], TL[89]; BA2,3,42,43(postCG)[[90]](#_ENREF_82), BA4[[90]](#_ENREF_82), BA6[[90]](#_ENREF_82), BA9[[90]](#_ENREF_82), BA10[[90]](#_ENREF_82), BA13[[90]](#_ENREF_82), BA18, 22(STG)[[90]](#_ENREF_82), BA30[[90]](#_ENREF_82), 40(IPL)[[90]](#_ENREF_82) |  |
| **ST44 (L)** | **Bil.** amACC(BA24,32)[[46]](#_ENREF_38), SMC(BA6)[[46]](#_ENREF_38), Th[[46]](#_ENREF_38), IN[[46]](#_ENREF_38), SII(BA40,43)[[46]](#_ENREF_38), IPL[45]; **Ipsi**. SI(BA3,1,2)[[46]](#_ENREF_38), postCG[45], Th[45], IN[45]; **Con**. STG[45] | **Bil.** Fpole(BA10)[[46]](#_ENREF_38), PCC(BA31)[[46]](#_ENREF_38), Cun(BA18,19)[[46]](#_ENREF_38); **Con.** Tpole(BA38)[[46]](#_ENREF_38); **Ipsi**. Amyg[[46]](#_ENREF_38), H[[46]](#_ENREF_38), preCun(BA7)[[46]](#_ENREF_38) |
| **SJ3 (L)** | **Bil.** SMA[91,92], TL[91,92], OL[91,92], FL[91,92], Ce[91,92], PL[91,92], Put[91,92]; **Con**. postCG[91,92], CingG[91,92]; |  |
| **SJ5 (Uni)** | **Bil.** FL[33,61,93,108], PL[33,61,93], TL[33,61,108], OL[33,61,108], H[33,61], CingG[33,61], basal ganglia[33,61], P[33,61], Ce[33,61,93,108], Th[108], PPL[108]; **Con**. TL[93], preCG[108], postCG[108], CingG[108], CI[108] |  |
| **SJ6 (R)** | **Bil.** FL[31,64], PL[31,64], TL[31]; **Con**. OL[64], TL[64] |  |
| **SJ8 (Bil)** | **Right** IFG(BA44,46)[[29]](#_ENREF_23) |  |
| **PC7 (R)** | **Con.** IFG(BA46,44)[[114]](#_ENREF_105), postCG[[114]](#_ENREF_105), IPL(BA40)[[114]](#_ENREF_105), STG(BA22)[[114]](#_ENREF_105); **Ipsi**. IFG(BA47,9)[[114]](#_ENREF_105), MFG(BA6)[[114]](#_ENREF_105) |  |
| **KI3 (Bil)** | **Unclear side:** BA22,29,38(STG), BA13(IN), BA4(preCG), BA3,43(postCG)[[115]](#_ENREF_106) | **Unclear side:** BA18, BA3(preCG), BA19(MOG), BA22(IN), BA39[[115]](#_ENREF_106) |
| **KI3 (R)** | **Bil.** postCG(BA2,3)[73]; **Ipsi.** STG(BA22)[73]; **Con**. MFG (BA46)[73], IFG(BA45)[73], IPL(BA40)[73]; **Unclear side**: AUC[[87]](#_ENREF_79); BA10(MFG), BA22,42(STG), BA43, 44(postCG)[[115]](#_ENREF_106) | **Unclear side:** BA6(SFG, preCG)[[115]](#_ENREF_106) |
| **KI6 (R)** |  | **Unclear side:** motor area[116,117] |
| **KI7 (NA)** | **Unclear side:** BA9(SFG,MFG)[[118]](#_ENREF_109), BA19(MTG,FG,IOG)[[118]](#_ENREF_109), BA23, 31, 32(post. CingG)[[118]](#_ENREF_109), BA24(ant. CingG)[[118]](#_ENREF_109), BA39(MTG)[[118]](#_ENREF_109) |  |
| **KI8 (NA)** | **Unclear side:** Cun(BA17/18/19)[[119]](#_ENREF_110), LG(BA17/18)[[119]](#_ENREF_110), preCun(BA31)[[119]](#_ENREF_110), preCG[[119]](#_ENREF_110), IFG[[119]](#_ENREF_110), SPG(BA7/22)[[119]](#_ENREF_110), MTG(BA21)[[119]](#_ENREF_110), ITG[[119]](#_ENREF_110), PCC[[119]](#_ENREF_110), culmen[[119]](#_ENREF_110); IOG(BA17/18)[[119]](#_ENREF_110), MOG(BA18)[[119]](#_ENREF_110), FG(BA20/37)[[119]](#_ENREF_110), lat. IN(BA13)[[119]](#_ENREF_110); **left** paraHG[[119]](#_ENREF_110) |  |
| **GB34 (Uni)** | **Bil.** PMC(BA6)[[34]](#_ENREF_28), SPL(BA7)[[34]](#_ENREF_28), IPG(BA40)[[37]](#_ENREF_31), Ce[[37]](#_ENREF_31), BA4,6,7(141); **Con.** OL[112], preCG(BA4)[[37]](#_ENREF_31), dor. FG(BA6)[[37]](#_ENREF_31), STG(BA41/42, 38,22)[[37]](#_ENREF_31), postCG(BA1,2)[[37]](#_ENREF_31), MEOC(BA18)[[37]](#_ENREF_31), Hyp[[37]](#_ENREF_31), cau[[37]](#_ENREF_31). ACC(BA24,32)[[37]](#_ENREF_31), Th[[37]](#_ENREF_31), MFG(BA10)[[37]](#_ENREF_31); **Ipsi.** M1(BA4)[[34]](#_ENREF_28), postCG(BA2)[[37]](#_ENREF_31), MFG(BA9,46)[[37]](#_ENREF_31), IN[[37]](#_ENREF_31); **Unclear side**: OL[106], FL[106], TL[106], basal ganglia[106], P[106]; | **Bil**. paraHG[[105]](#_ENREF_97), H[[105]](#_ENREF_97), CingG[[105]](#_ENREF_97), preCun[[105]](#_ENREF_97), Ce[[105]](#_ENREF_97); **Con.** rostral ACC(BA24,32)[[37]](#_ENREF_31) |
| **GB37 (NA)** | **Unclear side:** Cun(BA17/18/19)[[119]](#_ENREF_110), LG(BA17/18)[[119]](#_ENREF_110), preCun(BA31)[[119]](#_ENREF_110), preCG[[119]](#_ENREF_110), SFG[[119]](#_ENREF_110), MFG[[119]](#_ENREF_110), IFG[[119]](#_ENREF_110), SPG(BA7/22)[[119]](#_ENREF_110), MTG(BA21)[[119]](#_ENREF_110), ITG[[119]](#_ENREF_110), PCC[[119]](#_ENREF_110), culmen[[119]](#_ENREF_110), CingC(BA24/32)[[119]](#_ENREF_110), H[[119]](#_ENREF_110), Amyg[[119]](#_ENREF_110); OL[106], FL[106], TL[89,106], basal ganglia[106], P[106], IN[89] , V1[89]; **left** paraHG[[119]](#_ENREF_110) | **Bil**. Cun[[40]](#_ENREF_8), calcarine fissure[[40]](#_ENREF_8), LG[[40]](#_ENREF_8), lat. OG[[40]](#_ENREF_8) |
| **GB37 (Bil)** | **Unclear side:** SFG, MFG[113], IFG(BA10,11,45,47)[113]; STG(BA38)[113]; Cun[113], LG(BA18.19)[113] |  |
| **GB37 (Uni)** | **Bil.** Fop[[39]](#_ENREF_33), IN[[39]](#_ENREF_33), SII[[39]](#_ENREF_33); **Con.** MEFG[[39]](#_ENREF_33), OL[112], Basal ganglia[112]; **Ipsi**. SI[[39]](#_ENREF_33), IPL[[39]](#_ENREF_33); | **Bil**. MOPFC[[39]](#_ENREF_33), LG[[39]](#_ENREF_33), SPL[[39]](#_ENREF_33); **ispi**. paraCL[[39]](#_ENREF_33), postCG[[39]](#_ENREF_33), MTG[[39]](#_ENREF_33) |
| **GB43 (NA)** | **Bil.** A1[[43]](#_ENREF_35) | **Bil**. paraHG[[105]](#_ENREF_97), H[[105]](#_ENREF_97), CingG[[105]](#_ENREF_97), preCun[[105]](#_ENREF_97), Ce[[105]](#_ENREF_97) |
| **UB60 (R)** | **Bil.** Fop[[39]](#_ENREF_33), IN[[39]](#_ENREF_33), Ce[[39]](#_ENREF_33); **Ipsi**. SII[[39]](#_ENREF_33), MEFG[[39]](#_ENREF_33) | **Bil.** MOPFC[[39]](#_ENREF_33), Hyp[[39]](#_ENREF_33), Cun[[40]](#_ENREF_8), calcarine fissure[[40]](#_ENREF_8), LG[[40]](#_ENREF_8), lat. OG[[40]](#_ENREF_8); **Con**. paraH[[39]](#_ENREF_33); **Ipsi**. paraCL[[39]](#_ENREF_33), postCG[[39]](#_ENREF_33), MTG[[39]](#_ENREF_33) |
| **LR1 (L)** | **Bil.** STG[84], Ce[84]; **Con.** LG[84], SMG[84]; **Ipsi.** preCG[84], Cun(BA18)[84]; CC[84] |  |
| **LR2 (L)** | **Bil.** amACC(BA24,32)[[46]](#_ENREF_38), SMC(BA6)[[46]](#_ENREF_38), Th[[46]](#_ENREF_38), IN[[46]](#_ENREF_38), SII(BA40,43)[[46]](#_ENREF_38), CingG[84], Ce[84], IPL[45], IFL[45], Th[45]; **Con.** inf. OL[84], FG[84]; **Ipsi.** SI(BA3,1,2)[[46]](#_ENREF_38), SMG[84], LG[84], STG(BA22)[84], SN[84], postCG[45], claustrum[45]; | **Bil.** Fpole(BA10)[[46]](#_ENREF_38), pgACC(BA32)[[46]](#_ENREF_38), H[[46]](#_ENREF_38), paraH[[46]](#_ENREF_38), PCC(BA31)[[46]](#_ENREF_38), Cun(BA18,19)[[46]](#_ENREF_38), rspPCC(BA29,30)[[46]](#_ENREF_38); **Ipsi**. Amyg[[46]](#_ENREF_38) |
| **LR4 (L)** | **Con**. SPL[84], preCun(BA19)[84], H[84], SN[84]; **Ipsi**. preCG(BA4,6)[84], Ce[84] |  |
| **LR5 (L)** | **Bil.** MFG[84]; **Con**. preCG(BA4,10)[84], STG[84]; **Ipsi**. IFG[84] |  |
| **SI3 (L)** | **Bil.** TL[92,120], FL[92,120], OL[92,120], Ce[92,120], PPL[92,120], post CG[120], CingG[120]; **Con.** postCG[92], CingG[92], preCG[92,120]; **Ipsi.** IN[92,120], SMA[92,120], Cau[92,120], Put[92,120]; |  |
| **SI6 (R)** | **Con.** SFG(BA10)[[121]](#_ENREF_112), ITG(BA37)[[121]](#_ENREF_112), MTG(BA37)[[121]](#_ENREF_112), IPL(BA40)[[121]](#_ENREF_112); **Ipsi**. IFG(BA45,46)[[121]](#_ENREF_112) |  |
| **DU15** | **Bil.** STG(BA22,42)[[29]](#_ENREF_23) |  |
| **DU20** | **Bil.** STG[122], right preCG[122], postCG[122], SMG[122], MTG[122], dors. Th[122], Cau[122] | **Unclear side:**MFG[122], SFG[122], sup. CingG[122], MTG[122] |
| **BL6 (R)** | **Bil.** STG[123]; **Ipsi.** SMG[123]; **Con.** Th[123], CingG[123], paraHG[123], MFG[123],post.lobe of the Ce[123]; Interthalamic adhesion[123], habenular commissure of habenulae[123] | **Bil.** Ce[123], ITG[123]; Ipsi. IFG[123], midbrain[123] |
| **BL60 (L)** | **Unclear side:** OL[124], FL[124], ITL[124], Ce[124], VC[[87]](#_ENREF_79) |  |
| **BL62 (L)** | **Unclear side:** Ce[[125]](#_ENREF_116) |  |
| **BL67 (R)** | **Unclear side:** OL[124], FL[124], TL[124], Ce[124] |  |
| **HT7 (R)** | **Bil.** MFG[126], STG[126]; Con. postCG(BA2,1,43)[[121]](#_ENREF_112), postCG[126], pre CG[126], Th[126], Put[126], TTG[126], CingG[126], IPL(BA40)[[121]](#_ENREF_112), IFG(BA44)[[121]](#_ENREF_112), IN(BA40)[[121]](#_ENREF_112); **Ipsi.** IFG(BA47)[[121]](#_ENREF_112), STG(BA22)[[121]](#_ENREF_112) |  |

A1=primary auditory cortex, ACC=anterior cingulate cortex, AG=angular gyrus, amACC=anterior-middle anterior cingulate cortex, Amyg=Amygdala, ant.=anterior, Aq=aquaeductus cerebri, AUC=auditory cortex, BA=Brodmann area, BAGA=basal ganglia, Bil.=bilateral, BS=brainstem, cACCCau=caudate nucleus, CC=Corpus callosum, Ce=cerebellum, Cing=cingulate, Cing-am=anterior middle cingulate, CingC=cingulate cortex, CingG=cingulate gyrus, Cing-pm=posteromiddle cingulate, Cing-subgenu=Cingulate subgenual, Con.=contralateral, Cun=cuneus, dlPFC=dorsolateral prefrontal cortex, dm.=dorsomedial, dmPFC=dorsomedial prefrontal cortex, dors.=dorsal, FG=fusiform gyrus, FL=frontal lobe, Fop=frontal operculum, H=hippocampus, Hyp=hypothalamus, IFG=inferior frontal gyrus, IN=insula, inf.=inferior, infCol=inferior colliculi, IOG=inferior occipital gyrus, IPL=inferior parietal lobule, Ipsi.=ipsilateral, ITG=inferior temporal gyrus, L=left, lat.=lateral, LC=locus ceruleus, LG=lingual gyrus, LN=lenticular nucleus, M1=primary motor cortex, MEFG=medial frontal gyrus, MEOC=medial occipital cortex, MEOG=medial occipital gyrus, MFG=middle frontal gyrus, MOG=middle occipital gyrus, MOPFC=medial orbital prefrontal cortex, MTG=middle temporal gyrus, NA=not available, Nac=nucleus accumbens, NCF=nucleus cuneiformis, NRP=nucleus raphe pontis, NTS=nucleus tractus solitaries, O=operculum, OG=orbital gyrus, OL=occipital lobe, P=pons, PAG=periaqueductal gray, paraCL=paracentral lobule, paraH=parahippocampus, paraHG=parahippocampal gyrus, PBN=parabrachial nucleus, PC=parietal cortices , PCC=posterior cingulate cortices, PFC=prefrontal cortices, PFL=prefrontal lobe, pgACC=pregenual cingulate cortex, PL=parietal lobule, PMA=premotor area, PMC=premotor cortex, PN=pontine nuclei, PO=parietal operculum, post.=posterior, postCG=postcentral gyrus, PPL=posterior parietal lobe, preCG=precentral gyrus, preCun=precuneus, Put=putamen, PVN=paraventricular nucleus, R=right, rACC=rostral anterior cingulate cortex, RO=rolandic operculum, rspPCC=retrosplenial posterior cingulate cortex, RtTg=reticulo-tegmental, SFG=superior frontal gyrus, SI=primary somatosensory area, SII=second somatosensory area, SMA=supplementary motor area, SMC=supplementary motor cortex, SMG=supramarginal gyrus, SN=substantia nigra, SOG=superior occipital gyrus, SPL=superior parietal lobule, STG=superior temporal gyrus, sup.=superior, supCol=superior colliculi, Fpole=frontal pole, Th=thalamus, TL=temporal lobe, Tpole=temporal pole, Uni=unilateral, V1=primary visual cortices, VC=visual cortex, vl-=ventrolateral, vm-=ventromedial, vmPFC=ventromedial prefrontal cortex, VNC=vestibular nuclear complex.
